# Variation in stroke survivors’ long-term home care use: a South London population-based study

**DOI:** 10.1093/esj/aakag045

**Published:** 2026-05-11

**Authors:** Wasana Kalansooriya, Iain J Marshall, Mark McGlinchey, Eva S Emmett, Marina Soley Bori, Eleanor G V Stevens, Ajay Bhalla, David Wyatt, Sophie Rowland-Coomber, Yanzhong Wang, Abdel Douiri, Matthew D L O'Connell, Charles D A Wolfe, Julia Fox-Rushby

**Affiliations:** Department of Population Health Sciences, King’s College London, London, United Kingdom; Department of Population Health Sciences, King’s College London, London, United Kingdom; Department of Population Health Sciences, King’s College London, London, United Kingdom; Community Stroke and Neurorehabilitation Service, Guy’s and St Thomas’ NHS Foundation Trust, London, United Kingdom; Department of Population Health Sciences, King’s College London, London, United Kingdom; Department of Population Health Sciences, King’s College London, London, United Kingdom; Department of Population Health Sciences, King’s College London, London, United Kingdom; Department of Population Health Sciences, King’s College London, London, United Kingdom; Department of Ageing and Health, Guy’s and St Thomas’ NHS Foundation Trust, London, United Kingdom; Department of Population Health Sciences, King’s College London, London, United Kingdom; Department of Population Health Sciences, King’s College London, London, United Kingdom; Department of Population Health Sciences, King’s College London, London, United Kingdom; Department of Population Health Sciences, King’s College London, London, United Kingdom; Department of Population Health Sciences, King’s College London, London, United Kingdom; Department of Population Health Sciences, King’s College London, London, United Kingdom; Department of Population Health Sciences, King’s College London, London, United Kingdom

**Keywords:** home care, informal care, social care, stroke survivors, unmet needs

## Abstract

**Introduction:**

Policy shifts towards home-based care are reshaping the management of stroke survivors, many of whom require long-term support. Home care, which encompasses social care for personal and household tasks and informal care provided by family and friends, plays a crucial role in post-stroke recovery and community reintegration. This study examined home care use up to 15 years post-stroke and its associated factors, and assessed unmet needs for assistance with activities of daily living (ADLs).

**Patients and methods:**

Data from 7885 stroke survivors in the South London Stroke Register (1995–2022) were analysed at 3 months, 1, 5 and 15 years post-stroke. Descriptive analyses examined home care patterns and unmet needs. A Heckman selection model assessed factors associated with home care use while accounting for missing data.

**Results:**

On average, 75% of stroke survivors used home care across 15 years post-stroke, with 83% of care at 3 months and 87% at 15 years being provided through informal care. Home care use was more likely among those with functional dependency (29%; 95% CI, 22%–35%) and those living with family (24%; 95% CI, 21%–27%). Social care use was higher in people with greater dependency (48%; 95% CI, 42%–56%), those living alone (25%; 95% CI, 21%–29%), those with lower deprivation (10%; 95% CI, 1%–20%) or those with a Black ethnic background (6%; 95% CI, 2%–9%). Informal care use was higher among those living with family (12%; 95% CI, 9%–14%), those with moderate dependency (2%; 95% CI, 0.1%–5%) or females (6%; 95% CI, 3%–9%). Unmet needs in ADLs increased over time (12% at 3 months to 17% at 15 years) and were higher among those with moderate compared with severe functional dependency.

**Discussion and Conclusions:**

Within home care, informal care remains the predominant long-term support for stroke survivors, persisting up to 15 years after stroke. Addressing health-related, socio-economic, ethnic and gender disparities in home care and unmet needs is essential for equitable community-based stroke care, and caution is needed when promoting home-based care models regarding the distributional impact of home care. Accurate measurement of home care is key to improving post-stroke care models and quality of life.

## Graphical Abstract

**Graphical Abstract:**
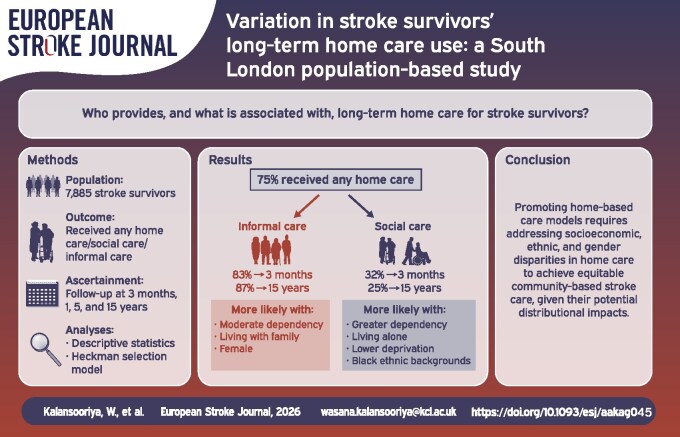

## Introduction

Despite advances in multidisciplinary stroke management,^1^ many stroke survivors experience long-term impairment: 39% have physical or cognitive limitations 5 years post-stroke,^2^ and 1 in 10 lives with moderate to severe disability at 15 years.^3,4^ As the global burden of cardiovascular disease increases,^5^ long-term care has become a critical priority.

Across Europe, policy emphasises accessible, high-quality long-term care,^6^ including post-stroke support.^7^ In the United Kingdom, reflecting international guidance promoting care in preferred residences,^8^ care is increasingly delivered at home.^9^ Home care, both social and informal care, includes personal care and household support,^10^ and is central to recovery and community reintegration.^11^ Social care shortages and reduced funding^12^ increase reliance on informal care, with the potential to exacerbate socio-economic inequalities.^13–16^ In the United Kingdom, unpaid care for stroke is valued at £15.8 billion annually, around 61% of total stroke-related costs,^17^ largely reflecting carers’ time, which raises concerns about the distributional impact of home-based care policies.

Research on home care is limited; it often relies on simplified assumptions,^18^ overlooks social care and focuses on either informal or formal care in isolation.^19–21^ Studies are typically based on small samples and short follow-up periods, and tend to prioritise carer outcomes.^22^ Although unmet care needs among stroke survivors are recognised, variations across demographic and socio-economic groups remain poorly understood.^23^

Using a large multiethnic population-based cohort, this study examines: (1) patterns of home care use up to 15 years post-stroke, (2) factors associated with overall home care use up to 5 years, (3) associations with the use of different types of care and (4) the presence of, and variations in, unmet home care needs. These findings will inform policy and commissioning of equitable, adequately funded long-term care services for stroke survivors and their families.

## Methods

### Data

Data were drawn from the South London Stroke Register (SLSR),^24^ an ongoing, population-based cohort recording first-ever stroke cases (1995–2022) using the World Health Organization’s ICD-10 criteria.^25^ Follow-up data at 3 months, 1, 5 and 15 years were analysed. These time points broadly align with the South London Stroke Register follow-up schedule,^24^ and were chosen to prioritise completeness and represent short-, medium-, long- and very long-term care use.

The study included community-dwelling stroke survivors (private or sheltered housing) and excluded those in residential or nursing care. At each follow-up, participants reported the receipt of help with personal care and household tasks (Appendix S1). Four outcomes were defined: (1) any home care use (social or informal care), (2) social care (home help/carer, voluntary organisations or meals on wheels), (3) informal care (family, friends or relatives) and (4) unmet needs (requiring help with at least 1 activity of daily living [ADL], but receiving no home care) (Appendix S2).

Variable selection was guided by established models of care utilisation,^14,26^ incorporating “need” and “predisposing and enabling” factors (Appendix S3). Need was operationalised as: stroke severity (NIHSS and the Glasgow Coma Scale), functional dependency (Barthel Index), multimorbidity (2 or more health conditions), cognitive status (abbreviated mental test), recurrent stroke and proximity to death. “Predisposing” and “enabling” factors included age at the first-ever stroke, gender, ethnicity, living arrangement, education, relative deprivation (index of multiple deprivation) and social support (Appendix S4a and S4b).

### Statistical methods

Descriptive and inferential statistics (*t*-tests, chi-square tests) were used to examine home care utilisation patterns up to 15 years post-stroke (objective 1). Factors associated with overall home care use (objective 2), were analysed using a Heckman selection model,^27,28^ chosen to address non-random attrition linked to unobserved determinants of follow-up participation, such as functional dependence. The first-stage (selection regression) models follow-up participation, and the second stage (outcome regression) estimates the probability of receiving home care using a linear probability model, adjusting for selection. Both stages include “need” and “predisposing and enabling” factors as explanatory variables, with the year of the stroke included in the selection regression to meet the exclusion criteria.

The same type of Heckman model was used to analyse social and informal care use separately (objective 3), modifying the selection equation to account for both follow-up participation and home care receipt. All regression analyses used cohort data from 3 months, 1 year and 5 years post-stroke. Unmet care needs (objective 4) were assessed using descriptive and inferential methods.

As sensitivity analyses,^29^ outcome regressions were re-estimated using complete cases and the multiple imputation chained equations method,^30^ including all main model variables. Further sensitivity analyses assessed alternative definitions of care receipt and unmet need (Appendices S1 and S2).

All analyses were conducted in Stata 18.^31^ Model fit was assessed using *R*^2,2^ Wald chi-square and F tests; cluster-robust standard errors accounted for heteroscedasticity and autocorrelation, and multicollinearity was checked using the variance inflation factor. Results are reported as associations, with only statistically significant findings at the 95% CI presented in the text.

## Results

### Sample characteristics

Of the 7885 stroke cohort members, 23%, 30%, 45% and 59% had died by 3 months, 1, 5 and 15 years, respectively (Table 5a of Appendix S5). Among survivors, follow-up completion rates were 65.7% (3 months), 70.6% (1 year), 64.9% (5 years) and 57.1% (15 years).

The study focused on community-dwelling survivors, with samples of 3344 (3 months), 3310 (1 year), 1839 (5 years) and 491 (15 years) (Table 5b of Appendix S5). Some survivors were missing at follow-up (Table 5a of Appendix S5), with non-random differences: younger, healthier and more independent individuals were more likely to be lost to follow-up (Appendix S6).

Among community-dwelling survivors, 42% had a minor stroke, 24% were fully dependent at 7 days (3% at 15 years), 28% had multimorbidity pre-stroke (58% at 15 years), 33% were cognitively impaired at the hospital (22% at 15 years) and the rate of recurrent stroke rose from 2% at 3 months to 8% at 15 years. The mean age at the first-ever stroke was 69, 54% were male, 58% lived with others, 54% were in the most deprived areas. By ethnic background, 64% were White, 28% were Black and 8% were in the “Other” category, which includes Asians (Appendix S8). Living arrangements changed for some survivors post-stroke: for example, at 3 months, 17% of those previously living alone moved in with others, while 11% transitioned to living alone (Appendix S9).

### Patterns of home care utilisation

**Figure 1:**
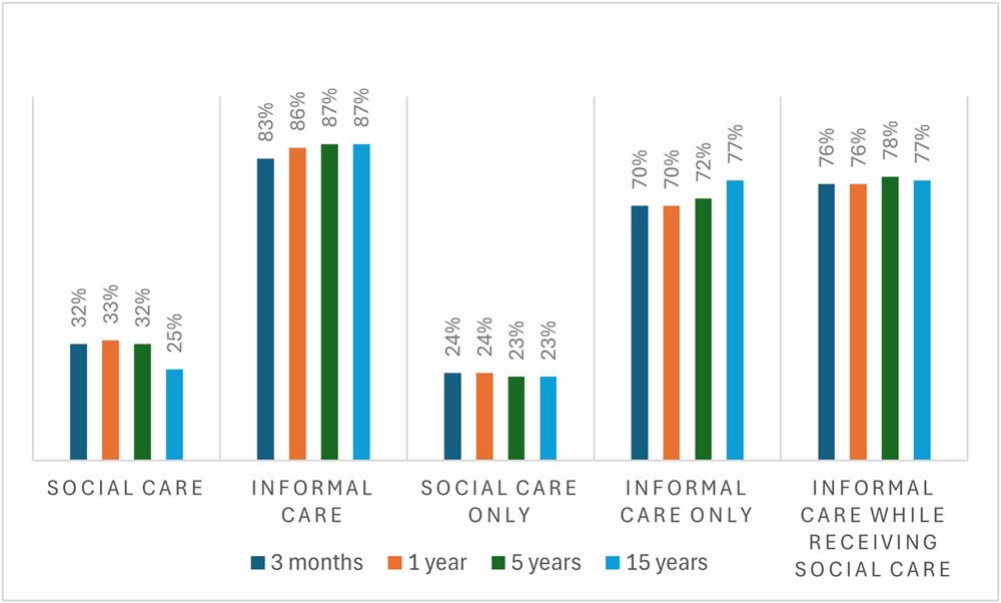
Bar chart showing the percentage of home care received through social care and/or informal care from 3 months to 15 years post-stroke.

Seventy-five percent of stroke survivors received some home care at 3 months, with little change up to 15 years (Appendix S10). Among survivors who received any home care, 32% accessed social care at 3 months (25% at 15 years), and 83% received informal care (87% at 15 years). Some survivors received both types of care; for example, at 3 months, 76% of those receiving social care also received informal care (Figure 1).

**Figure 2:**
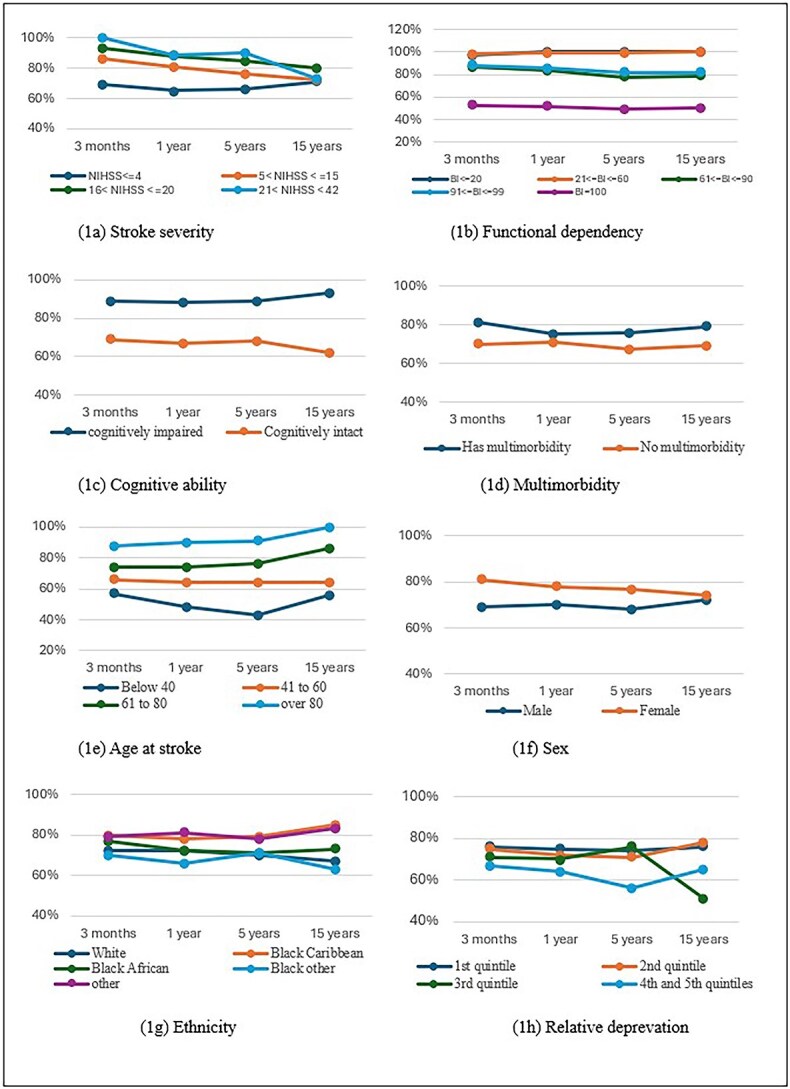
Line graphs showing the percentage of home care (any) received from 3 months to 15 years post-stroke by health-related, socio-economic and demographic characteristics.

Receipt of any home care varied by need (health status) and predisposing/enabling factors (socio-economic and demographic) (Figure 2 and Appendix S10). At 3 months, all survivors with a severe stroke, 97% of those who were functionally dependent, 81% with multimorbidity and 89% with cognitive impairment received home care; similar patterns were seen at 1, 5 and 15 years. Home care was also more common after a recurrent stroke. Survivors were consistently more likely to receive home care across follow-ups if they were older (88%), female (81%), of a Black Caribbean (80%) or “other” (79%) ethnic background, had no formal education (87%), lived with others (80%) or had someone to help (78%).

Receipt of different care types also varied by health, socio-economic and demographic factors (Appendix S11). Care type varied by proximity to death as survivors who died within 5 years were more likely to receive early home care, particularly social care, than those who survived for 5 years (Appendix S12).

### Factors associated with the use of any type of home care

**Figure 3:**
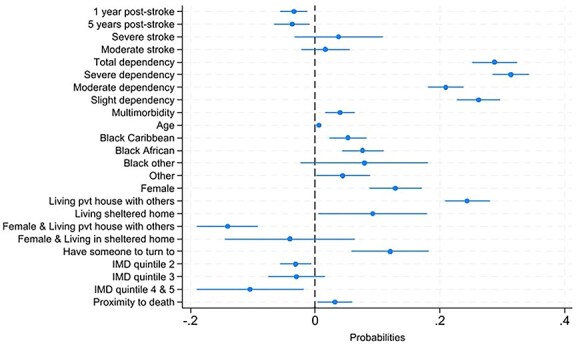
The figure shows the coefficients of the linear probability regression estimated through the Heckman selection model for the use of any home care (n=5157). Each coefficient is adjusted for other independent variables included in the model. The full result table is given in Table 1.

Home care use declined slightly over time, being 4% lower at 1 year and 5 years post-stroke than at 3 months. Home care use was associated with need and predisposing/enabling factors (Figure 3; Table 1).

**Table 1:** Factors affecting home care use up to 5 years post-stroke: results of the Heckman selection model.

			Receiving any home care	Receiving any social care if use home care	Receiving any informal care if use home care
	**Outcome regression: dependent variable: receiving home care**				
	Follow up (ref: 3 months)				
		1-year post-stroke	−0.034^**^	0.01	0.020^*^
		5-year post-stroke	−0.037^*^	−0.013	0.015
**Need factors**	Stroke severity (ref: mild stroke)				
		Severe stroke	0.037	0.129^*^	−0.020
		Moderate stroke	0.016	0.097^**^	−0.015
	Level of disability (ref: independent)				
		Total dependency	0.288^**^	0.471^**^	−0.135^**^
		Severe dependency	0.314^**^	0.358^**^	−0.059^**^
		Moderate dependency	0.210^**^	0.149^**^	0.019^*^
		Slight dependency	0.262^**^	0.034	0.023
	Multimorbidity		0.040^*^	−0.007	0.004
	Proximity to death		0.041^*^	−0.060^*^	0.019
**Pre-disposing and enabling factors**	Age at initial stroke		0.006^**^	0.003^**^	0.000
	Ethnicity (ref: White)				
		Black Caribbean	0.052^**^	0.056^*^	0.024^*^
		Black African	0.076^**^	0.046^*^	0.002
		Black other	0.079	−0.064	0.021
		Other	0.044^*^	0.061^*^	0.029^*^
	Gender (female = 1)		0.129^**^	−0.034	0.058^**^
	Living arrangements (ref: living in private house alone)				
		Private house (with others)	0.244^**^	−0.255^**^	0.125^**^
		Sheltered home	0.092^*^	0.063	0.003
	Gender ^*^ living arrangements (ref: male^*^ living in private hose alone)				
		Female^*^ private house (with others)	−0.140^**^	0.102^**^	−0.046^*^
		Female^*^ sheltered home	−0.041	0.075	0.002
	Have someone to turn to		0.121^**^	−0.168^**^	0.303^**^
	Relative deprivation (ref: most deprived -first quintile)				
		Second quintile	−0.031^*^	−0.014	−0.013
		Third quintile	−0.029	0.011	−0.020
		Fourth and fifth quintiles	−0.105^*^	0.106^*^	−0.029
	cons		−0.117	0.457^**^	0.499^**^
**Selection model: dependent variable: completed the follow-up or missing**					
		Stroke severity	0.235^**^	0.291^**^	0.254^**^
		Barthel index (7 day)	0.049^**^	−0.172^**^	−0.138^**^
		Pre-stroke multimorbidity	0.027	0.171^**^	0.156^*^
		Age at stroke	0.011^**^	0.020^**^	0.021^**^
		Ethnicity	0.004	0.049^**^	0.046^**^
		Female	−0.001	0.103^*^	0.103^*^
		IMD quint	0.024	−0.048	−0.058
		Stroke year	0.041^**^	0.023^**^	0.029^**^
		Pre-stroke living condition	−0.011	0.102^**^	0.121^**^
		_cons	−83.81.^**^	−48.0^**^	−58.48^**^
**Regression diagnostics**	var(error. Home care use)		0.108	0.112	0.056
	corr(error include in followup ^*^ error home care use)		0.081	−0.567^**^	0.112^*^
	var(home care use[id])		0.037	0.068	0.004
	var(Include in follow up[id])		0.876	0.852	0.706
	corr(include in followup ^*^ home care use[id])		0.223^**^	−0.262^**^	0.162
	Number of observation in the selection regression		8,389	8,389	8,389
	Number of observation in the selected regression		5,157	3,863	3,863
	Wald chi2(23)		1,269.93	847.57	450.28

Abbreviation: IMD, index of multiple deprivation.

^**^Significant at 1% significance level ^*^Significant at 5% significance level. The table displays the estimated coefficient of the Heckman selection model, which accounts for missing follow-up data. Panel data for the 3 follow-up periods are used. The selection regression is a probit model with a dummy-dependent variable indicating whether the respondent is included in the follow-up or is missing. The total number of observations considered is 14,859 (Table 5a of Appendix S5), but only 8389 observations are used due to item missingness. Outcome regressions are linear probability models with the dependent variable (1) receiving any home care (*n* = 5157), (2) receiving social care (*n* = 3863) and (3) receiving informal care (*n* = 3863).

Among need factors, functional dependency was strongly associated with home care use: severely dependent survivors were 31% more likely, and moderately dependent survivors 21% more likely, to receive home care than those who were independent. Multimorbidity and proximity to death were also associated with slightly increased home care use.

Among predisposing/enabling factors, living with family was strongly associated with home care use, increasing the likelihood of receiving home care by 24%. Females were 13% more likely than males to receive home care; however, when living with others, they were 14% less likely to do so. Living in deprived areas was associated with a 10% higher probability of receiving home care, and survivors of Black Caribbean (5%), Black African (7%) and “Other” (4%) ethnic backgrounds were more likely to receive home care than White survivors.

### Factors associated with the use of different types of home care

**Figure 4:**
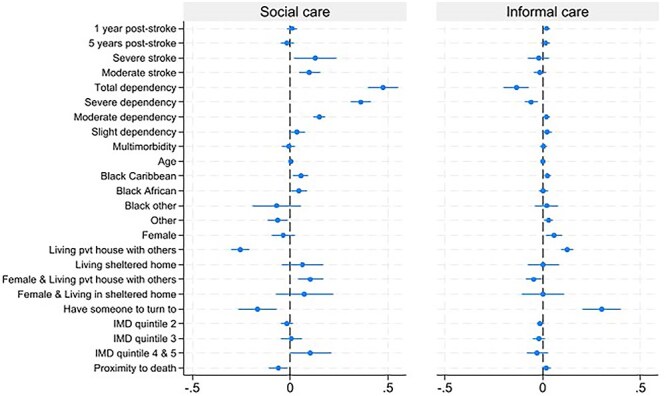
The figure shows the coefficients of the linear probability regressions estimated through the Heckman selection model for different care types; social care and informal care (n=3863). Each coefficient is adjusted for other independent variables included in the model. The full results table is given in Table 1.

Care type was associated with need factors (Figure 4; Table 1). Completely dependent survivors were 48% more likely to receive social care and 14% less likely to receive informal care than independent survivors. Survivors who had a severe stroke were 13% more likely than those with a mild stroke to use social care. Proximity to death was associated with reduced use of social care among those who had received any home care.

Care was also related to pre-disposing factors (Figure 4; Table 1). Survivors living with others were 26% less likely to receive social care and 12% more likely to receive informal care. The likelihood of informal care use was 30% higher, and social care was 17% lower, when someone was available to help. In general, women were 6% more likely than men to receive informal care; among those living with others, women were 10% more likely to receive social care and 5% less likely to receive informal care than men. Living in the least deprived areas increased the likelihood of receiving social care by 10%. Survivors with Black Caribbean and Black African ethnic backgrounds were 5%–7% more likely to use social care, while those with “Other” ethnic backgrounds were 6% less likely to use social care compared to those of a White ethnic background; additionally, survivors with Black Caribbean backgrounds were 2%, and those with “other” ethnic backgrounds were 3%, more likely to use informal care.

### Unmet needs of home care for ADLs

Table 2 shows the unmet ADL needs of survivors. At 3 months post-stroke, 46% of stroke survivors needed help with ADLs, decreasing slightly to 42% at 15 years. Unmet ADL needs rose from 12% at 3 months to 17% at 15 years. Some received home care (24% at 3 months and 17% at 15 years), and even social care (18% at 3 months and 10% at 15 years), despite having no ADL needs.

**Table 2:** Percentage of stroke survivors with unmet needs up to 15 years and socio-demographic and health-related differences of unmet needs from 3 months to 5 years post-stroke.

		3 months	1 year	5 years	15 years
**Sample size (*n*)**		3325	2587	1829	490
**Need care for ADLs**		1523 (45.8%)	1474 (44.7%)	817 (44.7%)	204 (41.6%)
**Need care for ADLs and receive home care**		1335 (88%)	1266 (86%)	663 (82%)	169 (83%)
**Need care for ADLs but do not receive home care (unmet needs)**		182 (12%)	205 (14%)	149 (18%)	35 (17%)
**Do not need care for ADLs, but receive home care**		439 (24%)	380 (21%)	193 (19%)	50 (17%)
**Need care for ADLs and receive social care**		698 (49%)	668 (48%)	351 (46%)	74 (40%)
**Need care for ADLs, but do not receive social care**		738 (51%)	725 (52%)	409 (54%)	112 (60%)
**Do not need care for ADLs but receive social care**		184 (18%)	176 (17%)	70 (13%)	17 (10%)
		**% with unmet needs for ADLs by socio-demographic and health-related differences**			
**Stroke severity**					
**Severe stroke**		4.6^*^	0.0^**^	7.1	20.0
**Moderate stroke**		7.1^*^	4.4^**^	20.2	15.8
**Mild stroke**		13.2^*^	15.7^**^	18.6	16.8
**Functional dependency**					
**Total dependency**		3.0^**^	0.0^**^	0.0^**^	0.0^**^
**Severe dependency**		2.4^**^	2.3^**^	3.2^**^	2.1^**^
**Moderate dependency**		11.3^**^	14.6^**^	22.5^**^	23.7^**^
**Slight dependency**		37.0^**^	35.7^**^	47.5^**^	20.0^**^
**Multimorbidity**					
**No**		13.5^*^	14.3	22.7^**^	23.7
**Yes**		10.5^*^	13.5	15.6^**^	13.3
**Age at stroke**					
**Less than 40**		13.3^**^	12.8^**^	16.7^**^	10.0
**40–60**		15.2^**^	19.2^**^	26.2^**^	25.3
**61–80**		13.7^**^	16.3^**^	18.6^**^	11.6
**Above 81**		7.6^**^	5.8^**^	4.7^**^	12.5
**Gender**					
**Male**		14.5^**^	15.2	20.9	10.4^**^
**Female**		10.1^**^	12.8	15.7	24.5^**^
**Ethnicity**					
**White**		13.6	15.2	20.8	25.0^*^
**Black Caribbean**		11.0	12.9	15.3	9.1^*^
**Black African**		5.9	10.8	9.9	16.7^*^
**Black other**		9.5	15.8	26.7	0.0^*^
**Other**		11.5	11.6	18.8	0.0^*^
**Relative deprivation (IMD)**					
**First quintile**		12.9	13.3	16.5^**^	16.4
**Second quintile**		10.3	14.0	18.1^**^	16.7
**Third quintile**		10.1	16.8	25.5^**^	25
**Fourth and fifth quintiles**		17.8	11.9	43.5^**^	50
**Living condition**					
**Private household alone**		14.1	15.9	21.3	19.1
**Private household with others**		11.0	13.4	17.4	14.1
**Sheltered home**		11.0	10.1	15.1	38.5

Abbreviations: ADLs, activities of daily living; IMD, index of multiple deprivation.

The first part of the table presents the proportion of stroke survivors with home care needs and with unmet needs. Home care needs are identified based on the inability or need for help to do at least 1 of the 7 ADLs (see Appendix SS2). The second part of the table shows the proportion of stroke survivors with unmet needs according to health-related and socio-economic categories, as opposed to those who meet their care needs. Differences between the 2 groups were tested using the chi-squared and the Wilcoxon rank sum tests. Differences are significant at: ^**^ 1% significance level, ^*^ 5% significance level.

Unmet needs were higher among those with a mild stroke (13%), the slightly dependent (37%), those with no multimorbidity (14%) and younger survivors (17%). Men reported unmet need at 3 months (14%), while more women had unmet needs at 15 years (24%). Those in less deprived areas (at 5 years) had more unmet needs (44%), and White survivors at 15 years (25%) recorded more unmet ADL needs (Table 2).

### Sensitivity analyses

Sensitivity analyses largely mirrored the main results. Differences in the significance of stroke severity may reflect the pattern of missing data (Appendices S13 and S14). Using an alternative home care variable showed similar results, except for the insignificant coefficient for living conditions (Appendix S15). Unmet needs were lower when considering care for 2 or more ADLs (Appendix S16).

## Discussion

This study examined long-term patterns of home care use and its associated factors up to 15 years post-stroke, using data from a long-term, ethnically diverse, population-based stroke register. It is the first mapping of extended home care trajectories, demonstrating how functional disability, multimorbidity and social and demographic factors—rather than initial stroke severity—are related to long-term care needs.

Although many survivors continue to receive care in the long-term, home care use is highest shortly after the initial stroke, likely reflecting greater disability, emotional support needs, end-of-life care and post-discharge care arrangements. Informal care often increased 1-year post-stroke, suggesting growing reliance on family support as survivors adapt to long-term disability.

Functional dependency emerged as the strongest associate of home care, increasing alongside multimorbidity and the growing complexity of needs. Survivors with greater functional disability were more likely to receive social care rather than informal care, contrary to earlier findings linking disability primarily to informal care.^20,32,33^ While this may indicate that formal social care systems are appropriately targeting individuals with greater needs, survivors with severe disability often experience multi-domain challenges compounded by unclear responsibilities, limited resources and fragmented care pathways,^34^ meaning gaps and inequalities in provision can still persist despite this targeting.

Living arrangement is associated with home care use, with higher use among those living with others, largely related to household assistance such as cooking and cleaning. When these tasks are excluded, sensitivity analysis shows no statistically significant association. Higher care use among those living with others suggests some survivors may be unable to live alone post-stroke, consistent with findings that some moved in with others post-stroke. In contrast, a few survivors transitioned from living with others to living alone, although no statistically significant differences in their care arrangements could be detected due to the small sample size.

Ethnic disparities in home care use were evident. Survivors with Black Caribbean and Black African ethnic backgrounds were more likely to receive home care, particularly social care, than those of White ethnicity, possibly reflecting longer post-stroke survival combined with poorer functional outcomes in these groups.^35^ Black Caribbean survivors were also more likely to receive informal care. The “Other” ethnic group (including Asians) relied less on social care, possibly reflecting a greater reliance on informal care to meet their needs. These ethnic variations may be driven by sociocultural factors, such as multigenerational households and strong family caregiving norms, including familism and the cultural suitability of services, which are particularly common in Asian and African cultures.^36,37^ Language barriers between paid carers and survivors, and difficulties in accessing rehabilitation resources and service information, may also help explain the lower social care use and greater reliance on informal care within these communities.^36^

Gender differences were also apparent. Overall, women were more likely than men to receive home care, consistent with their higher levels of post-stroke impairment.^38^ However, when living with others, women were less likely than men to receive home care, suggesting potential inequalities in household support. When women did receive care, it was more often from social care than informal sources, consistent with gendered caregiving roles in which women are typically caregivers rather than care recipients.^39^

Socio-economic disparities were also notable. Survivors residing in more deprived areas received more overall home care, likely due to the increased provision of informal care in multigenerational households. However, they were less likely to receive social care, suggesting barriers related to affordability, access and lower social capital,^40^ compounded by limited funding for social care.^12^ Further research could examine how reliance on informal care affects the financial and economic well-being of caregivers in deprived communities.

Patterns of substitution and complementarity between social and informal care were observed. Survivors living alone relied mainly on social care, while those living with others depended primarily on informal care. However, survivors with moderate functional disability or of Black Caribbean ethnicity were more likely to receive both types of support, indicating some complementarity in addressing practical and emotional needs. This complementarity was not observed for gender. Women living with family were more likely to receive social care and less likely to receive informal care, highlighting concerns about how women’s emotional support needs are met, as these are often provided through informal care.^41^

Unmet needs for ADLs increased over time, aligning with prior studies showing persistent and emerging care needs.^23^ These unmet needs were higher among younger survivors and those with moderate disabilities, suggesting that care systems may inadequately serve groups perceived as relatively independent. Some survivors received social care without reported ADL difficulties, possibly reflecting unmeasured needs related to instrumental activities of daily living (IADLs) or emotional support, or potentially an inefficient allocation of social care.

These findings have several policy implications for Europe and the United Kingdom. With stroke events projected to rise,^42^ the European Stroke Organisation’s long-term care plan calls for stronger, sustained care pathways post-discharge.^7,43^ Some European countries have yet to provide a life-after-stroke programme, and care plans should include periodic reassessment of needs,^7^ not just discharge evaluation. In the United Kingdom, needs-based rehabilitation, within the Integrated Community Stroke Service (ICSS) could optimise outcomes and resource use. While social care appears better targeted to those with severe disability, addressing inequities in care access by ethnicity and social deprivation should be a priority when shifting hospital-centred care to community and home-based care.^9^ Care strategies should also account for multimorbidity, which increases long-term care needs.^44^ Unmet needs among younger people and those with moderate disabilities indicate that some groups fall outside existing support thresholds, highlighting a gap that should be addressed. Gender disparities in informal caregiving, especially the low level of informal care received by women in family settings, further highlight the need to strengthen social care for women and imply caution against home-based care policies that could leave them unsupported.

Future research should enhance data collection to fully operationalise the conceptual framework, enabling more accurate assessment of care needs, use and outcomes over time. Applying explicit causal frameworks could clarify pathways linking survivor characteristics to care use. Detailed information on care hours and funding could address gaps in service provision and financing,^45^ supporting more coordinated and equitable care. Investigating unmet care needs including care needs for IADLs, socio-economic and demographic inequalities and the effectiveness of combinations of formal, social and informal care, along with a detailed assessment of home care, would enable a more in-depth evaluation of how variation in home care affects outcomes, efficiency and equity. This could inform targeted interventions and optimise quality of life across the post-stroke trajectory.

The study has several limitations. While we were able to address missing follow-up data using appropriate econometric methods, follow-up missingness is common in long-term cohorts and the limited number of follow-up points prevented us from detecting potential serial correlation.^46^ Information on formal healthcare services received at home, income, household composition and community-level variables were unavailable, limiting the ability to fully operationalise the conceptual model outlined in Appendix S3. Intensity and funding of home care were not captured in the data, and care given was not related to functional needs. Unmet needs were assessed only for ADLs, as data on neither IADLs nor needs for IADLs or emotional support were collected, although such care needs exist and are related to home care use.^47^

With respect to the generalisability of our findings, we are aware that our study population is more ethnically diverse than the general population. While this implies the need for some caution in applying results nationally, rather than a weakness, we see this as a strength as much health research underrepresents ethnic minorities, especially from Black African diaspora communities.^48^ The weaknesses of synthetic oversampling methods, shown especially when machine learning algorithms are applied to minority groups using real-world data,^49^ and the importance the National Institute for Health Research places on ensuring equality, diversity and inclusion is embedded in research to ensure improvements in health nationally,^50^ mean it is especially important to have a good-quality data relevant to minority groups. Our detailed findings could be used to improve both representation and future models of long-term care for stroke patients. Patterns of home care use may also vary across regions depending on demographics, healthcare systems and social care arrangements. In England, social care is allocated by local authorities through means-tested, needs-based assessments under standardised statutory guidance,^51,52^ suggesting that these findings are generalisable to other local authorities within England, assuming needs are similar. Caution is warranted, however, when applying these findings to countries with different social care systems.

## Conclusion

Long-term home care is widespread and requires careful assessment and prioritisation. Recognising health and socio-economic disparities, variations in the use of different types of home care, and unmet needs is crucial for shaping effective policies, and caution should be taken regarding the distributional impact of home care when promoting home-based care models. Limitations in measuring home care highlight the need for better-quality data. Improved measurement would deepen understanding of the complex interactions among home-based services and help identify the most effective and equitable combinations of care to enhance the quality of life for stroke survivors and their families.

## Supplementary Material

aakag045_Appendix_(revised)
