# Supplementary material for: Variation in stroke survivors’ long-term home care use: a South London population-based study
Source: Eur Stroke J. 2026 May 11;11(5):aakag045. doi: 10.1093/esj/aakag045 (PMC13160425; doi:10.1093/esj/aakag045)
Supplement: aakag045_Appendix_(revised) [file aakag045_appendix_(revised).docx]

**Appendices**

Table of contents

|  |  | Page number |
| --- | --- | --- |
| Appendix 1 | Questions related to resource use for home care in the SLSR questionnaire | 1 |
| Appendix 2 | Identifying unmet needs of home care | 2 |
| Appendix 3 | Conceptual framework | 3 |
| Appendix 4a | Explanatory variables used | 4 |
| Appendix 4b | Hypothesis | 5 |
| Appendix 5 | SLSR population, follow-up status and missing in follow-up | 6 |
| Appendix 6 | Differences between complete and missing data | 7 |
| Appendix 7 | Item-level missingness for each variable | 8 |
| Appendix 8 | Sample characteristics | 9 |
| Appendix 9 | Changes in living conditions of stroke survivors between each follow-up period of cohort | 11 |
| Appendix 10 | Receiving any home care by health-related and socio-demographic variables | 12 |
| Appendix 11 | Types of home care by health -related and socio-demographic variables | 13 |
| Appendix 12 | Home care use with proximity to death | 14 |
| Appendix 13 | Factors affecting home care use up to 5 years post-stroke: Complete case analysis | 15 |
| Appendix 14 | Factors affecting home care use up to 5 years post-stroke: Analysis with multiple imputations | 17 |
| Appendix 15 | Sensitivity analysis: Factors affecting home care use (if required) and the main type of home care | 18 |
| Appendix 16 | Sensitivity analysis: An Alternative approach to identify unmet needs | 19 |
|  |  |  |
|  |  |  |

Appendix 1: Questions related to resource use for home care in the SLSR questionnaire


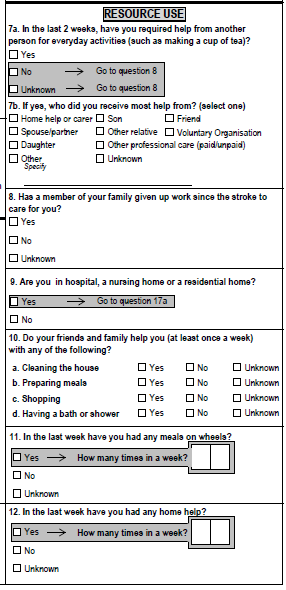


|  | Variable | Related questions |
| --- | --- | --- |
| (1) | Receive any home care | Yes to Q7a, 8, 10, 11,12 |
| (2) | Receive any social care | ‘home help or carer’ and ‘voluntary organisations’ in Q7b, Yes to Q11 or 12 |
| (3) | Receive any informal care | ‘Spouse/partner’, ‘Daughter’, ‘Son’, ‘Other relative’, ‘Friend’ in Q7b, Yes to Q8, Q10 |
| (4) | Unmet needs | See Appendix 2 |
| (5) | Receive home care (only if main source is recorded) | Yes to Q7a, 11,12 |
| (6) | Main type of care is social care | ‘home help or carer’ and ‘voluntary organisations’ in Q7b |
| (7) | Unmet needs(a) | See Appendix 2 |

Appendix 2: Identifying unmet needs for home care

Unmet needs for home care cannot be identified directly in the SLSR questionnaire. The Activities of Daily Living (ADLs) and Instrumental Activities of Daily Living (IADLs) can be used to identify need.^1,2^ Although there is no pre-agreed threshold for identifying care needs, previous research has considered an individual's difficulties in at least one to three ADLs or IADLs as indicative of care needs.^1–3^

The SLSR collects data for ADLs using the Barthel Index. This assessment measures independence in performing 10 ADLs (feeding, bathing, grooming, dressing, bowel, bladder, and toilet use, transfers, mobility, stairs). Only seven of these ten tasks (feeding, bathing, grooming, dressing, bowel, bladder, and toilet use) are routinely undertaken by paid carers funded by social services. The other three ADL items (transfers, mobility, stairs) are less likely to be undertaken by paid carers funded by social services. Therefore, if a stroke survivor is unable to do or needs help with one or more of the seven ADLs and does not receive any home care, we consider it an ‘unmet need’.

In sensitivity analysis, we included all 10 ADL items. If a stroke survivor is unable to do or needs help to do two or more of the 10 ADLs and does not receive any home care, it is identified as an unmet need.

Appendix 3: Conceptual framework


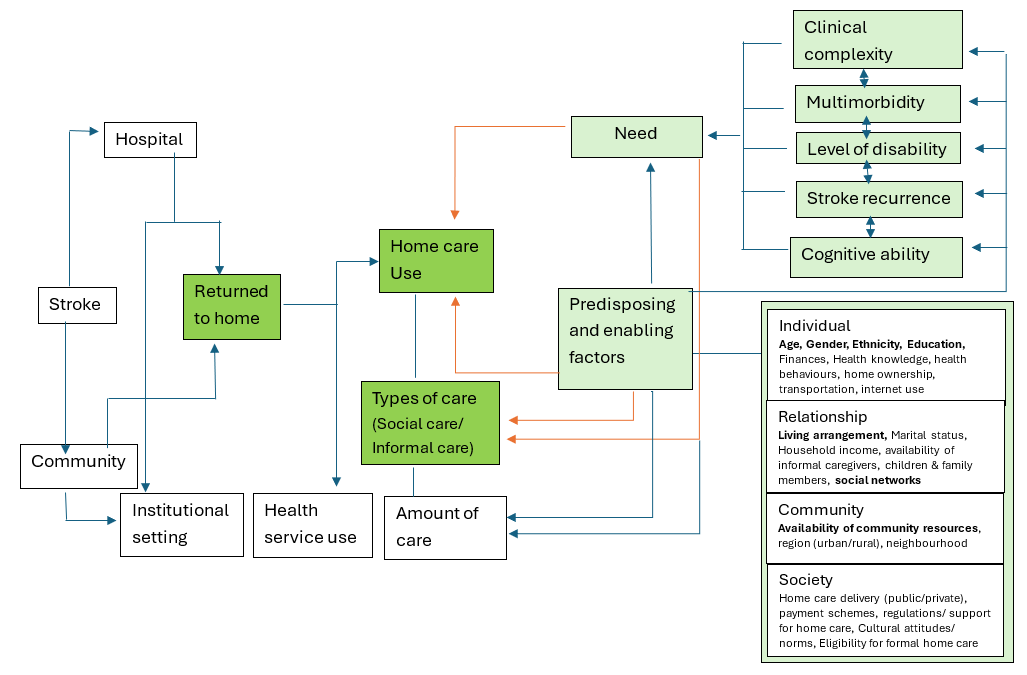


Notes: The conceptual framework shows the factors that influence resource utilization for home care once stroke survivors return home after a stroke. This framework combines two models: the healthcare utilization model developed by Andersen et al (1995) and the model proposed by Mah et al. (2021) as a socio-ecological model for homecare in community-dwelling older adults. According to the Andersen model, home care use is influenced by care needs and predisposing and enabling factors. Need factors are linked with current health conditions and the level of disability of stroke survivors. The clinical complexity of the stroke, presence of other health conditions, recurrence of stroke, and cognitive ability influence the health status of a stroke survivor at the follow-up stage. The Mah et el. model is embedded within the model to describe pre-disposing and enabling factors that affect home care utilisation. It includes individual factors such as age, gender, ethnicity, education, finances, health knowledge, and health behaviours, etc; relationship factors such as living arrangements, marital status, having children, etc; community-level factors such as availability of community resources, neighbourhood, etc; and society level factors such as public/private provision of homecare, payment and support schemes, cultural attitudes, etc.

All types of variables were searched for within the SLSR and any available were included. This model is also used to highlight the limitations of the data with suggestions for future research described in the discussion.

Andersen, R. M. (1995). Revisiting the behavioral model and access to medical care: Does it matter? *Journal of Health and Social Behavior*, 1–10.^4^

Mah, J. C., Stevens, S. J., Keefe, J. M., Rockwood, K., & Andrew, M. K. (2021). Social factors influencing utilization of home care in community-dwelling older adults: A scoping review. *BMC Geriatrics*, *21*(1), 145.^5^

Appendix 4: Explanatory variables used

| Variable | Description |
| --- | --- |
| Stroke severity* | Mild = (NIHSS<=4)  Moderate = (5< NIHSS < =15)  Moderate to severe = (16< NIHSS < =20)  Severe= (21< NIHSS < 42) |
|  | Severe = (GCS<=8)  Moderate = (9 <= GCS <=12)  Mild = (13<= GCS <=15) |
| Functional dependency | Total dependency= (BI<=20)  Severe dependency = (21<=BI<=60)  Moderate dependency = (61<=BI<=90)  Slight dependency= (91<=BI<=99)  Independent= (BI=100) |
| Multimorbidity | 1= Having 2 or more other health conditions  0= less than 2 or no other health conditions |
| Cognitive status | 1 = Cognitively impaired (0-7 AMT and <24 MMSE)  0 = Cognitively intact (8-10 AMT and 24+ MMSE) |
| Recurrent stroke | 1= stroke recurrence 0= No stroke recurrence |
| Proximity to death | 1= Died before the next follow-up  0 = Not died by the next follow-up |
| Age at first stroke | Years |
| Gender | 1= Female 0= Male |
| Ethnicity | 1= White 2= Black Caribbean  3= Black African 4= Black other 5= Other  (subcategories of Black ethnic groups are included, as their proportions are high within this population) |
| Living arrangement | 1= Private house alone 2= Private house with others  3= Sheltered home |
| Education | 1= No education 2= Up to primary education  3= Up to secondary education 4= Tertiary education |
| Relative deprivation  (measured using IMD) | 1= 1^st^ quintile (most deprived) 2= 2^nd^ quintile  3= 3^rd^ quintile 4= 4^th^ & 5^th^ quintiles ( least deprived)  (4th and 5th quintiles were combined due to the small number of participants in each group) |
| Social relations | 1= Has someone to turn to when need help  0 = Has no one to turn to when need help |

Abbreviations: NIHSS- National Institutes of Health Stroke Scale, GCS- Glasgow Coma Scale, BI- Barthel Index, AMT- Abbreviated Mental Test, MMSE- Mini-Mental State Examination

*Stroke severity is measured using NIHSS in the descriptive analysis. Due to the high number of missing data, it was not used in the regression analysis. Instead, GCS was used.

Appendix 4b: Hypotheses

|  | Variables included in the model | Hypothesis | Supporting evidence |
| --- | --- | --- | --- |
| Need Factors | Stroke severity | The higher the stroke severity higher the home care use | All these are ‘need’ factors. Anderson’s behavioural model explicitly shows that need factors are the core determinants of whether individuals use health services. We apply the same for home care use as Care needs and use are strongly associated with the severity of the health conditions, disability level, and proximity to death.^6–9^ |
|  | Functional disability | The higher the functional disability, higher the home care use |  |
|  | Multimorbidity | Having multimorbidity is positively associated with home care use |  |
|  | Proximity to death | Proximity to death is positively associated with home care use |  |
| Predisposing and enabling factors | Age at stroke | Older age is positively associated with home care use | Mah et al^5^ report that 88% of the studies they reviewed show that older age is positively associated with home care use. |
|  | Gender | Women use more home care than men | Mah et al^5^ report that 14 of 39 studies found that women use more home care than men, and an equal number found no significant difference.  Women experienced higher survival and significantly poorer outcomes in activities of daily living than men during post-stroke. |
|  | Ethnicity | Stroke survivors in Minority ethnic groups use more homecare than those of White ethnicity. | Mah et al^5^ report a borderline association between ethnicity and home care use. Cultural factors affect home care use among minority ethnic groups, increasing care received from family members.^10,11^ People from minority ethnic backgrounds are more likely to provide high-intensity, unpaid care within the home.^11^ |
|  | Living arrangement | Living with family is positively associated with home care use, particularly informal care. | Mah et al^5^ report an uncertain relationship between living arrangement and home care use as studies show positive or no association between living and home care use. However, they show that living alone is associated with formal home care services, and co-residents typically provide informal care that may substitute for, delay, or reduce formal home care use. Studies show that co-resident caregiving tends to be more intensive.^12^ |
|  | Social status (Relative deprivation) | The higher the deprivation, the higher the home care use, particularly from informal care | Socioeconomically disadvantaged areas were associated with earlier needs for home support.^13^ Functional recovery after stroke is poor in the deprived population. People in more deprived areas were more likely to receive informal home care.^14^ |
|  | Social relationship (Having someone to turn to when needed help) | Having someone to turn to when help is needed is positively associated with home care use, particularly informal care. | Mah et al^5^) report that social relationships (networks, household, marital status) are significant factors shaping home care use patterns; however, the effects depend on their availability and willingness to provide informal care. Social networks are associated with increased access to informal care among older adults.^15^ |

Appendix 5: Sample sizes: whole and selected SLSR population at each follow-up point, with numbers and reasons for missing data

Table 5a: Follow-up status of stroke survivors

|  | | **3-month**  **follow-up** | | **1-year**  **follow-up** | | **5-year**  **follow-up** | | **15-year**  **follow-up** | |
| --- | --- | --- | --- | --- | --- | --- | --- | --- | --- |
|  |  | ***Number of stroke survivors*** | ***%*** | ***Number of stroke survivors*** | ***%*** | ***Number of stroke survivors*** | % | ***Number of stroke survivors*** | % |
| Completed | | 3,995 | 50.7 | 3,873 | 50.5 | 2,139 | 27.1 | 561 | 7.1 |
| Died | | 1,806 | 22.9 | 2,264 | 29.5 | 3,529 | 44.8 | 4,662 | 59.2 |
| Not reached the follow-up | |  |  | 138 | 1.8 | 1,059 | 13.4 | 2,241 | 28.4 |
| Missing | | 2084 | 26.4 | 1610 | 18.2 | 1158 | 14.7 | 421 | 5.3 |
|  | Late notification* | *1,057* | *13.4* | *441* | *5.8* | *107* | *1.4* | *5* | *0.1* |
|  | Emigrated | *27* | *0.3* | *48* | *0.6* | *77* | *1.0* | *68* | *0.9* |
|  | Refused | *60* | *0.8* | *93* | *1.2* | *150* | *1.9* | *135* | *1.7* |
|  | Lost for other reasons | *940* | *11.9* | *1028* | *10.6* | *824* | *10.5* | *213* | *2.7* |
| **Total** | | **7,885** | **100** | **7,885** | **100** | **7,885** | **100** | **7885** | **100** |
|  | | | | | | | | | |
| **Follow-up is expected for (Total – (Died + not reached the follow-up))** | | **6,079** |  | **5,483** |  | **3,297** |  | **982** |  |
| % completed | |  | 65.7 |  | 70.6 |  | 64.9 |  | 57.1 |
| % Missing | |  | 34.3 |  | 29.4 |  | 35.1 |  | 42.9 |

*stroke survivors who recorded their initial stroke after the post-stroke follow-up.

Table 5b: Sub Sample (Stroke survivors living in private house or sheltered home)

|  | **3-month**  **follow-up** | | **1-year**  **follow-up** | | **5-year**  **follow-up** | | **15-year follow-up** | |
| --- | --- | --- | --- | --- | --- | --- | --- | --- |
| **In private house or sheltered home** | **3344** | **84%** | **3310** | **85%** | **1839** | **86%** | **491** | **88%** |
| In Residential home/ nursing home/hospital | 613 | 15% | 439 | 11% | 230 | 11% | 43 | 8% |
| Missing data for the living place | 38 | 1% | 124 | 3% | 70 | 3% | 27 | 5% |
| Total SS completed the follow-up | 3995 |  | 3873 |  | 2139 |  | 561 |  |

Table 5a: Out of 7885 stroke patients, 51%, 50%, 27%, and 7% completed 3-month, 1-year, 5-year, and 15-year follow-ups. The percentage of deaths increases from 23% to 59% from 3 months to 15 years. Some stroke survivors had not reached their follow-up stage (eg. 28% at 15 years). Stroke survivors are missing in the follow-ups due to; late notification of the stroke incidence, emigration from the area, refusal to participate or some other reasons. Accordingly, 26% are missing at 3 months, and this decreases to 5% missing at 15 years. After excluding stroke survivors who died or who had not reached the follow-up, data should be available for 6079 stroke survivors at 3 months. However, 66% completed the follow-up, and 34% are missing at 3 months, rising to 43% at 15 years.

Table 5b: The analysis used stroke survivors living in private houses and sheltered homes at the follow-up stage. 84% of stroke survivors who completed the 3 months follow-up were living in private/sheltered homes.

Appendix 6: Differences in patient characteristics for those with complete vs. missing data

|  | **3-month follow-up** | | **1-year follow-up** | | | **5-year follow-up** | | | **15-year follow-up** | |
| --- | --- | --- | --- | --- | --- | --- | --- | --- | --- | --- |
|  | Completed the follow-up | Missing data | Completed the follow-up | Missing data | Completed the follow-up | | Missing data | Completed the follow-up | | Missing data |
| **Number of stroke survivors** | **3995** | **2084** | **3873** | **1610** | **2139** | | **1158** | **561** | | **421** |
|  | **66%** | **34%** | **71%** | **29%** | **65%** | | **35%** | **57%** | | **43%** |
| **Stroke severity** |  | |  | | |  | | |  | |
| Severe | 3.3 | 3.9 | 3.0** | 4.8** | 3.3 | | 4.4 | 4.4 | | 5.0 |
| Moderate | 10.2 | 10.0 | 9.2** | 10.9** | 9.0 | | 8.7 | 7.3 | | 7.4 |
| Mild | 86.5 | 86.1 | 87.8** | 84.3** | 87.7 | | 86.9 | 88.3 | | 87.6 |
| **Barthel index (7day)** |  | |  | | |  | | |  | |
| Total dependency | 16.3** | 11.5** | 13.5** | 12.2** | 10.5 | | 11.8 | 11.1* | | 15.5* |
| Severe dependency | 23.1** | 18.2** | 22.0** | 18.3** | 17.9 | | 18.1 | 16.4* | | 20.6* |
| Moderate dependency | 23.3** | 19.3** | 22.7** | 19.6** | 21.4 | | 18.3 | 18.0* | | 20.1* |
| Slight dependency | 4.1** | 4.0** | 4.3** | 4.1** | 5.0 | | 3.7 | 4.6* | | 4.3* |
| Independence | 33.1** | 47.0** | 37.5** | 45.7** | 45.3 | | 48.1 | 49.8* | | 39.5* |
| **Having Multimorbidity (pre stroke)** | | |  | | |  | | |  | |
| Yes | 30.4** | 23.9** | 28.8** | 22.3** | 19.8 | | 17.7 | 9.8 | | 8.6 |
| No | 69.5** | 76.1** | 71.2** | 77.7** | 80.2 | | 82.3 | 90.2 | | 91.4 |
| **Cognitive status (at hospital** | | |  | | |  | | |  | |
| Impaired | 30.2* | 26.5* | 28.3 | 27.2 | 23.8 | | 23.8 | 20.1** | | 30.8** |
| Intact | 69.7* | 73.5* | 71.7 | 72.8 | 76.2 | | 76.2 | 79.9** | | 69.2** |
| **Age at stroke** |  | |  | | |  | | |  | |
| below 40 | 4.1** | 7.6** | 4.4** | 8.6** | 5.6** | | 10.9** | 12.3** | | 16.4** |
| 41-60 | 22.4** | 28.1** | 23.8** | 31.1** | 30.3** | | 33.2** | 43.5** | | 36.8** |
| 61-80 | 49.1** | 45.3** | 50.1** | 43.8** | 51.1** | | 46.2** | 42.4** | | 42.3** |
| above 81 | 24.2** | 19.0** | 21.7** | 16.5** | 13.0** | | 9.7** | 1.8** | | 4.5** |
| **Gender** |  | |  | | |  | | |  | |
| Male | 53.2** | 57.1** | 54.9 | 57.2 | 56.1 | | 58.8 | 55.8 | | 58.2 |
| Female | 46.7** | 42.9** | 45.1 | 42.8 | 43.9 | | 41.2 | 44.2 | | 41.8 |
| **Ethnicity** |  | |  | | |  | | |  | |
| White | 60.8 | 58.6 | 60.2** | 57.2** | 59.4 | | 57.0 | 55.0 | | 55.0 |
| Black Caribbean | 16.1 | 16.3 | 16.9** | 15.4** | 16.4 | | 16.9 | 19.5 | | 14.9 |
| Black African | 13.8 | 14.7 | 13.7** | 16.6** | 14.8 | | 15.8 | 16.1 | | 17.8 |
| Black other | 1.2 | 1.4 | 1.2** | 1.2** | 1.4 | | 0.6 | 1.4 | | 1.5 |
| Other | 8.0 | 9.0 | 8.0** | 9.7** | 8.0 | | 9.7 | 6.0 | | 10.8 |
| **Living conditions (pre-stroke)** | | |  | | |  | | |  | |
| Private household alone | 34.7 | 33.8 | 33.9 | 33.6 | 34.0 | | 31.5 | 23.7 | | 28.1 |
| Private household shared | 58.8 | 61.2 | 60.4 | 61.9 | 62.2 | | 64.0 | 73.1 | | 69.1 |
| Sheltered home | 3.9 | 3.1 | 3.6 | 2.9 | 2.3 | | 2.8 | 1.8 | | 1.4 |
| Residential/Nursing/ | 1.6 | 1.2 | 1.4 | 1.0 | 0.6 | | 0.7 | 0.6 | | 0.3 |
| Hospital | 0.1 | 0.1 | 0.1 | 0 | 0.1 | | 0 | 0.2 | | 0 |
| Other | 0.6 | 0.6 | 0.6 | 0.6 | 0.8 | | 1.0 | 0.6 | | 1.1 |
| **IMD quintile** |  | |  | | |  | | |  | |
| 1st quintile | 51.4 | 53.2 | 50.6** | 55.6** | 54.7 | | 58.2 | 59.1 | | 63.3 |
| 2nd quintile | 37.1 | 37.0 | 37.9** | 35.0** | 34.6 | | 30.8 | 30.4 | | 24.7 |
| 3rd quintile | 8.2 | 7.2 | 8.1** | 6.8** | 7.1 | | 6.8 | 7.2 | | 6.0 |
| 4th & 5th quintiles | 3.3 | 2.6 | 3.4** | 2.6** | 3.6 | | 4.2 | 3.3 | | 6.0 |

** Significant differences between groups exist 1% significance level, * 5% significance level

Appendix 6 illustrates the differences between stroke survivors who did and did not participate in the follow-up. Differences were tested using Chi-Square test and Wilcoxon Rank Sum tests. At 3 months post-stroke, significant differences can be observed between those who did and did not provide data regarding functional dependency, multimorbidity, cognitive status, age, and gender. At 1 year post-stroke, these differences extend to stroke severity and level of deprivation, while the gender difference disappears. At 5 years post-stroke, significant differences occur for age only. At 15 years post-stroke, the two samples are significantly different in terms of functional dependency and age.

Appendix 7: Item level missingness, by variable (Cohort data 3 months to 5 years)

|  |  | (1)  Number of observations completed follow-ups | | (2)  Number of observations  missed follow-ups | | (1) + (2)  Number of observations which the data should be available | |
| --- | --- | --- | --- | --- | --- | --- | --- |
|  |  | Data is available | Missing | Data is available | Missing | Data is available | Missing |
| Variables measured at initial stroke | NIHSS* | 5743 | 2750 | 3428 | 1424 | 9171 | 4174 |
|  | GCS | 8192 | 301 | 4428 | 424 | 12620 | 725 |
|  | Barthel index (7 day) | 7651 | 842 | 4044 | 808 | 11695 | 1650 |
|  | Multimorbidity (pre-stroke) | 6929 | 1564 | 4340 | 512 | 11269 | 2076 |
|  | Age at stroke | 8422 | 71 | 4759 | 93 | 13181 | 164 |
|  | Female | 8423 | 70 | 4763 | 89 | 13186 | 159 |
|  | Ethnicity | 8403 | 90 | 4672 | 180 | 13075 | 270 |
|  | IMD quintiles | 8068 | 425 | 4527 | 325 | 12595 | 750 |
|  | Living condition(pre-stroke) | 7789 | 704 | 4275 | 577 | 12064 | 1281 |
|  | Stroke year | 8493 | 0 | 4800 | 52 | 13293 | 52 |
|  | Education* | 5089 | 3404 | 2759 | 2093 | 7848 | 5497 |
|  |  |  |  |  |  |  |  |
| Variables captured at the follow-up stage | Barthel Index(follow-up) | 8310 | 183 | 0 | 4852 | 8310 | 5035 |
|  | Multimorbidity(follow-up) | 8450 | 43 | 0 | 4852 | 8450 | 4895 |
|  | Living condition (Follow-up) | 8493 | 0 | 0 | 4852 | 8493 | 4852 |
|  | Have someone to turn to | 7110 | 1383 | 0 | 4852 | 7110 | 6235 |
|  | Living condition* gender | 8423 | 70 | 0 | 4852 | 8423 | 4922 |
|  | Recurrent stroke* | 4776 | 3717 | 0 | 4852 | 4776 | 8569 |
|  | Cognitive ability* | 5953 | 2540 | 0 | 4852 | 5953 | 7392 |
|  | Proximity to death | 8493 | 0 | 4852 | 0 | 13345 | 0 |
|  | Receive home care (any)** | 8480 | 13 | 0 | 4852 | 8480 | 4865 |
|  | Social care(any)** | 8292 | 201 | 0 | 4852 | 8292 | 5053 |
|  | Informal care (any)** | 8067 | 426 | 0 | 4852 | 8067 | 5278 |
|  | Use informal care as the main help (If using home care)** | 3513 | 378 | 0 | 4852 | 3513 | 5230 |
|  | Use social care as the main help (if using home care)** | 3513 | 378 | 0 | 4852 | 3513 | 5230 |

Abbreviations: NIHSS- National Institutes of Health Stroke Scale, GCS- Glasgow Coma Scale, IMD: Index of Multiple Deprivation

* Variables used for descriptive analysis only. Due to a high number of missing data, these variables were not used in the regression analysis.

** Dependent variables of the study

This table shows the item-level missing observations for each variable for the 3 time periods. The total sample was divided into two groups: (1) those who completed follow-ups and lived in private or sheltered homes and (2) those who missed follow-ups. For each variable, 8,493 observations should be available for the group (1) (3,344 at 3 months, 3,310 at 1 year, and 1,839 at 5-year follow-ups), and 4852 observations should be available for group (2) (2084 at 3 months, 1610 at 1 year and 1158 at 5 years follow-ups). In total, each variable should have 13345 observations.

Appendix 8: Sample characteristics, by follow-up point of cohort

| Variable | **At stroke** | **3-month follow-up** | **1 Year** | **5 Years** | **15 Years** |
| --- | --- | --- | --- | --- | --- |
|  | **n=7885** | **n=3344** | **n=3310** | **n=1839** | **n=491** |
| **Stroke severity (NIHSS)** |  |  |  |  |  |
| Minor | 42% | 51% | 52% | 55% | 53% |
| Moderate | 39% | 42% | 40% | 38% | 40% |
| Moderate to Severe | 8% | 5% | 5% | 5% | 3% |
| Severe | 10% | 2% | 3% | 2% | 3% |
| **Stroke severity (GCS)** |  |  |  |  |  |
| Severe | 12% | 2% | 2% | 3% | 4% |
| Moderate troke | 12% | 7% | 7% | 8% | 7% |
| Mild | 74% | 90% | 89% | 88% | 88% |
| **Functional dependency (Barthel index)*** |  |  |  |  |  |
| Total dependency | 25% | 4% | 3% | 3% | 3% |
| Severe dependency | 20% | 11% | 10% | 11% | 10% |
| Moderate dependency | 19% | 35% | 36% | 45% | 43% |
| Slight dependency | 4% | 10% | 11% | 8% | 12% |
| Independent | 32% | 41% | 40% | 34% | 32% |
| **Multimorbidity (yes)** | 28% | 43% | 51% | 59% | 58% |
| **Cognitive status (intact)** | 67% | 78% | 76% | 76% | 78% |
| **Recurrent stroke (yes)** |  | 2% | 4% | 4% | 8% |
| **Age at stroke** | 69.1 | 67.6 | 66.6 | 63.4 | 55.6 |
|  | (15.3) | (14.4) | (14.3) | (13.8) | (12.8) |
| **Gender (female)** | 48% | 46% | 44% | 43% | 43% |
| **Ethnicity** |  |  |  |  |  |
| White | 64% | 60% | 59% | 59% | 55% |
| Black Caribbean | 15% | 16% | 17% | 16% | 18% |
| Black African | 12% | 14% | 14% | 15% | 17% |
| Black other | 1% | 1% | 1% | 2% | 2% |
| Other* | 8% | 8% | 8% | 8% | 8% |
| **Relative deprivation (IMD)** |  |  |  |  |  |
| 1st quintile | 54% | 50% | 51% | 55% | 60% |
| 2nd quintile | 36% | 38% | 38% | 35% | 30% |
| 3rd quintile | 7% | 8% | 8% | 7% | 7% |
| 4th quintile | 2% | 3% | 3% | 3% | 3% |
| 5th quintile | 0% | 1% | 1% | 1% | 1% |
| **Living arrangement** |  |  |  |  |  |
| Private house (alone) | 35% | 35% | 35% | 36% | 37% |
| Private house (with others) | 58% | 61% | 60% | 58% | 59% |
| Sheltered home | 4% | 4% | 5% | 6% | 4% |
| Residential/nursing/care home/ Hospital* | 3% |  |  |  |  |
| **Education** |  |  |  |  |  |
| No formal education | 2% | 2% | 2% | 2% | 1% |
| Primary | 8% | 8% | 8% | 6% | 4% |
| Secondary | 75% | 76% | 76% | 75% | 82% |
| Tertiary | 15% | 14% | 15% | 17% | 14% |
| **Employment** |  |  |  |  |  |
| Retired | 67% | 63% | 62% | 66% | 71% |
| Unable to work | 6% | 22% | 19% | 17% | 11% |
| Full time | 18% | 6% | 7% | 9% | 9% |
| Part time | 3% | 4% | 5% | 4% | 5% |
| unemployed | 4% | 3% | 5% | 3% | 2% |
| Other | 2% | 1% | 2% | 1% | 1% |
| **Social relations (Have someone to turn to= yes)** |  | 97% | 97% | 96% | 97% |

Abbreviations: NIHSS- National Institutes of Health Stroke Scale, GCS- Glasgow Coma Scale, IMD: Index of Multiple Deprivation

The characteristics of the sample at five different time points are given in the table. 42% of initial strokes were minor as per NIHSS (74% are mild according to GCS). This increased to 53% for those followed up to 15 years. 32% were entirely functionally dependent 7 days post-stroke, and decreased to 4% at 15 years post-stroke. 28% had multimorbidity before stroke, rising to 59% at 5 years. 33% had cognitive impairment in the hospital, increasing to 78% at 15 years. 2% had a recurrent stroke at 3 months, rising to 8% at 15 years. The mean age at the initial stroke was 69 years (SD=15). 48% were female. 64% belonged to the white ethnic group, and this percentage decreased to 55% for the sample 15 years post-stroke. 61% lived in a private house with others at 3 months, and it declined to 59% at 15 years.

*Categorisation of ethnicity in the SLSR changed over the course of the time (from 1995 to the present). In the early years (before 2007, all minority ethnic groups, excluding Black ethnicities, were categorised as ‘Other’. Since 2007/08, more detailed ethnic categories are available, and Asians 5% of the population in SLSR. Therefore, Asians are likely to represent a large portion of the ‘Other’ ethnic category prior to 2007. To maximise data on ethnicity, we had to maintain consistency of the variable across time, and therefore used the variable where Asians are categorised in the ‘Other’ category.’

Appendix 9: Changes in living conditions of stroke survivors between each follow-up period of cohort

**1: Private household alone, 2: Private household with others, 3: Sheltered home, 4: Residential/Nursing/care home, 5: Hospital, 6: Other**

|  |  | **Living condition at 3 months post-stroke** | | | |  |  | |  | **Living condition at 1 year post-stroke** | | | |
| --- | --- | --- | --- | --- | --- | --- | --- | --- | --- | --- | --- | --- | --- |
|  |  | *1* | *2* | *3* | *Total* |  |  | |  | *1* | *2* | *3* | *Total* |
| **Pre-stroke living condition** | *1* | 824 | 183 | 43 | 1,050 |  | **Living condition at 3 months post-stroke** | | *1* | 642 | 140 | 35 | 817 |
|  | *%* | 78.48 | 17.43 | 4.1 | 100 |  |  |  | *%* | 78.58 | 17.14 | 4.28 | 100 |
|  | *2* | 207 | 1,692 | 12 | 1,911 |  |  |  | *2* | 195 | 1,268 | 19 | 1,482 |
|  | *%* | 10.83 | 88.54 | 0.63 | 100 |  |  |  | *%* | 13.16 | 85.56 | 1.28 | 100 |
|  | *3* | 29 | 8 | 63 | 100 |  |  |  | *3* | 12 | 8 | 63 | 83 |
|  | *%* | 29 | 8 | 63 | 100 |  |  |  | *%* | 14.46 | 9.64 | 75.9 | 100 |
|  | *4* | 5 | 1 | 1 | 7 |  |  |  | *4* | 14 | 49 | 9 | 72 |
|  | *%* | 71.43 | 14.29 | 14.29 | 100 |  |  |  | *%* | 19.44 | 68.06 | 12.5 | 100 |
|  | *5* | 1 | 2 | 0 | 3 |  |  |  | *5* | 11 | 17 | 2 | 30 |
|  | *%* | 33.33 | 66.67 | 0 | 100 |  |  |  | *%* | 36.67 | 56.67 | 6.67 | 100 |
|  | *6* | 5 | 4 | 4 | 13 |  |  |  | *6* | 9 | 24 | 5 | 38 |
|  | *%* | 38.46 | 30.77 | 30.77 | 100 |  |  |  | *%* | 23.68 | 63.16 | 13.16 | 100 |
|  | *Total* | 1,071 | 1,890 | 123 | 3,084 |  |  |  | *Total* | 883 | 1,506 | 133 | 2,522 |
|  |  | 34.73 | 61.28 | 3.99 | 100 |  |  |  | *%* | 35.01 | 59.71 | 5.27 | 100 |
|  | ***chi2 = 2.5e+03 Pr = 0.000*** | | | | |  |  | | ***chi2 = 2.0e+03 Pr = 0.000*** | | | | |
|  | | | | | |  | |  | | | | | |
|  |  | **Living condition at 5 year post-stroke** | | | |  |  | |  | **Living condition at 15 year post-stroke** | | | |
|  |  | *1* | *2* | *3* | *Total* |  |  | |  | *1* | *2* | *3* | *Total* |
| **Living condition at 1 year post-stroke** | *1* | 291 | 102 | 30 | 423 |  | **Living condition at 5 year post-stroke** | | *1* | 62 | 28 | 9 | 99 |
|  | *%* | 68.79 | 24.11 | 7.09 | 100 |  |  |  | *%* | 62.63 | 28.28 | 9.09 | 100 |
|  | *2* | 153 | 688 | 11 | 852 |  |  |  | *2* | 59 | 167 | 3 | 229 |
|  | *%* | 17.96 | 80.75 | 1.29 | 100 |  |  |  | *%* | 25.76 | 72.93 | 1.31 | 100 |
|  | *3* | 14 | 9 | 35 | 58 |  |  |  | *3* | 3 | 4 | 2 | 9 |
|  | *%* | 24.14 | 15.52 | 60.34 | 100 |  |  |  | *%* | 33.33 | 44.44 | 22.22 | 100 |
|  | *4* | 1 | 7 | 7 | 15 |  |  |  | *4* | 0 | 1 | 0 | 1 |
|  | *%* | 6.67 | 46.67 | 46.67 | 100 |  |  |  | *%* | 0 | 100 | 0 | 100 |
|  | *5* | 0 | 1 | 0 | 1 |  |  |  | *5* | 0 | 3 | 0 | 3 |
|  | *%* | 0 | 100 | 0 | 100 |  |  |  | *%* | 0 | 100 | 0 | 100 |
|  | *6* | 6 | 4 | 1 | 11 |  |  |  | *6* | 0 | 5 | 0 | 5 |
|  | *%* | 54.55 | 36.36 | 9.09 | 100 |  |  |  | *%* | 0 | 100 | 0 | 100 |
|  | *Total* | 465 | 811 | 84 | 1,360 |  |  |  | *Total* | 124 | 208 | 14 | 346 |
|  |  | 34.19 | 59.63 | 6.18 | 100 |  |  |  |  | 35.84 | 60.12 | 4.05 | 100 |
|  | ***chi2 = 743.4138 Pr = 0.000*** | | | | |  |  | | ***chi2 = 73.4134 Pr = 0.000*** | | | | |

Appendix 10: Receiving any home care by health-related and socio-demographic variables

|  |  | | **3 months** | | **1 year** | | **5 years** | | **15 years** | |
| --- | --- | --- | --- | --- | --- | --- | --- | --- | --- | --- |
|  |  |  | Number of obs. | *% Receive home care* | Number of obs. | *% Receive home care* | Number of obs. | *% Receive home care* | Number of obs. | *% Receive home care* |
|  | **All** | | 3337 | 74.77 | 3306 | 73.2 | 1837 | 72 | 484 | 72.5 |
| Need factors | Stroke severity | |  |  |  |  |  |  |  |  |
|  |  | Minor (NIHSS<=4) | 1,145 | 69.1** | 1,172 | 65.1** | 700 | 66.4** | 171 | 71.4 |
|  |  | Moderate (5< NIHSS < =15) | 932 | 86.3** | 898 | 80.5** | 479 | 75.6** | 129 | 72.1 |
|  |  | Moderate to Severe (16< NIHSS < =20) | 103 | 93.2** | 108 | 87.9** | 61 | 85.2** | 10 | 80 |
|  |  | Severe (21< NIHSS < 42) | 48 | 100** | 57 | 89.5** | 30 | 90** | 11 | 72.7 |
|  | Level of dependency | |  |  |  |  |  |  |  |  |
|  |  | Total dependency (BI<=20) | 133 | 96.9** | 102 | 100** | 47 | 100** | 16 | 100** |
|  |  | Severe dependency (21<=BI<=60) | 349 | 97.7** | 312 | 99.1** | 190 | 98.9** | 47 | 100** |
|  |  | Moderate dependency (61<=BI<=90) | 1,150 | 87.1** | 1,168 | 84.2** | 809 | 78.5** | 201 | 78.6** |
|  |  | Slight dependency (91<=BI<=99) | 312 | 87.8** | 349 | 85.9** | 139 | 82.1** | 55 | 81.8** |
|  |  | Independent (BI=100) | 1,331 | 53.1** | 1,306 | 51.5** | 600 | 49.2** | 151 | 49.7** |
|  | Multimorbidity | |  |  |  |  |  |  |  |  |
|  |  | Yes | 1,428 | 80.9** | 1,689 | 75.4** | 1071 | 75.5** | 148 | 79.1* |
|  |  | No | 1,890 | 70.1** | 1,603 | 70.9** | 756 | 66.9** | 193 | 69.4* |
|  | Cognitive status | |  |  |  |  |  |  |  |  |
|  |  | cognitively impaired | 566 | 88.9** | 524 | 88.2** | 289 | 88.6** | 55 | 92.7** |
|  |  | Cognitively intact | 1,969 | 68.7** | 1,701 | 66.6** | 896 | 67.6** | 199 | 62.3** |
|  | Recurrent stroke | |  |  |  |  |  |  |  |  |
|  |  | Yes | 38 | 84.2 | 64 | 85.9* | 53 | 79.2 | 38 | 86.8* |
|  |  | No | 1,738 | 76.9 | 1,661 | 71.3* | 1221 | 71.2 | 439 | 70.8* |
| Predisposing and enabling factors | Age categories | |  |  |  |  |  |  |  |  |
|  |  | Below 40 | 148 | 57.4** | 161 | 47.8** | 107 | 42.9** | 64 | 56.2** |
|  |  | 41 to 60 | 786 | 66.1** | 835 | 64.3** | 580 | 64.5** | 217 | 64.1** |
|  |  | 61 to 80 | 1,659 | 74.4** | 1,681 | 73.9** | 939 | 75.6** | 195 | 86.2** |
|  |  | over 80 | 742 | 88.3** | 627 | 89.8** | 211 | 90.5** | 8 | 100** |
|  | Gender | |  |  |  |  |  |  |  |  |
|  |  | Male | 1,796 | 69.1** | 1,824 | 69.7** | 1048 | 68.4** | 280 | 71.8 |
|  |  | Female | 1,508 | 81.4** | 1,458 | 77.6** | 776 | 76.6** | 204 | 73.5 |
|  | Ethnicity | |  |  |  |  |  |  |  |  |
|  |  | White | 1,970 | 72.2** | 1,932 | 71.6** | 1071 | 69.6* | 261 | 67.4* |
|  |  | Black Caribbean | 540 | 80** | 560 | 77.9** | 299 | 78.9* | 86 | 84.9* |
|  |  | Black African | 477 | 77.4** | 468 | 72.1** | 265 | 70.9* | 80 | 72.5* |
|  |  | Black other | 44 | 70.4** | 41 | 65.8** | 28 | 71.4* | 8 | 62.5* |
|  |  | other | 275 | 79.3** | 269 | 80.7** | 152 | 78.3* | 41 | 82.9* |
|  | Relative deprivation | |  |  |  |  |  |  |  |  |
|  |  | 1st quintile | 1,595 | 75.9 | 1,601 | 75.1* | 953 | 73.6* | 292 | 76.1** |
|  |  | 2nd quintile | 1,229 | 74.9 | 1,204 | 72.1* | 604 | 70.9* | 154 | 77.9** |
|  |  | 3rd quintile | 258 | 70.5 | 236 | 70.3* | 114 | 76.3* | 35 | 51.4** |
|  |  | 4th and 5th quintiles | 104 | 67.3 | 100 | 64* | 57 | 56.1* | 17 | 64.7** |
|  | Education | |  |  |  |  |  |  |  |  |
|  |  | No formal education | 38 | 86.8** | 31 | 80.6** | 23 | 91.3** | 1 | 100 |
|  |  | Primary | 157 | 83.4** | 155 | 81.9** | 69 | 81.2** | 7 | 85.7 |
|  |  | Secondary | 1,512 | 77.6** | 1,482 | 74.5** | 848 | 72.2** | 152 | 73.1 |
|  |  | Tertiary | 287 | 65.5** | 289 | 55.1** | 192 | 59.4** | 26 | 69.2 |
|  | Living condition | |  |  |  |  |  |  |  |  |
|  |  | Private house (alone) | 1,154 | 65.1** | 1,162 | 63.2** | 666 | 59.2** | 180 | 60** |
|  |  | Private house (with others) | 2,050 | 79.5** | 1,982 | 78.6** | 1068 | 78.8** | 286 | 79.7** |
|  |  | Sheltered home | 133 | 85.7** | 162 | 79.6** | 103 | 83.5** | 18 | 83.3** |
|  | Social relations (Have someone to turn to) | |  |  |  |  |  |  |  |  |
|  |  | Yes | 2,453 | 77.5** | 2,678 | 73.6** | 1725 | 72.2** | 465 | 73.1** |
|  |  | No | 85 | 56.5** | 96 | 55.2** | 67 | 56.7** | 14 | 57.1** |

The table shows the percentage of stroke survivors receiving home care in each category as opposed to those not receiving home care. Differences in receiving home care between the two groups were tested using the chi-squared and the Wilcoxon Rank Sum tests. Differences are significant at: ** 1% significance level, * 5% significance level

Appendix 11: Type of home care received, by health-related and socio-demographic variables

|  |  |  | **% receiving any level of social care if they receive any home care** | | | **% receiving any level of informal care if they receive any home care** | | |
| --- | --- | --- | --- | --- | --- | --- | --- | --- |
|  |  |  | 3 months | 1 year | 5 years | 3 months | 1 year | 5 years |
|  | All | | 32.3 | 33.3 | 31.7 | 83.2 | 86 | 87 |
| Need factors | Stroke severity | |  |  |  |  |  |  |
|  |  | Minor (NIHSS<=4) | 23.4** | 24.1** | 24.5** | 93.1* | 94.1 | 92.9 |
|  |  | Moderate (5< NIHSS < =15) | 38.7** | 39.5** | 37.5** | 92.4* | 93.5 | 94.4 |
|  |  | Moderate to Severe (16< NIHSS < =20) | 57.3** | 56.8** | 42.3** | 89.5* | 93.6 | 92.3 |
|  |  | Severe (21< NIHSS < 42) | 45.8** | 62.7** | 59.2** | 81.2* | 94.1 | 88.8 |
|  | Level of Disability | |  |  |  |  |  |  |
|  |  | Total dependency (BI<=20) | 72.9** | 80.3** | 76.6** | 72.8** | 92.1* | 82.9* |
|  |  | Severe dependency (21<=BI<=60) | 59.2** | 64.4** | 62.7** | 81.8** | 90.6* | 92.1* |
|  |  | Moderate dependency (61<=BI<=90) | 39.6** | 38.1** | 31.5** | 91.6** | 94.6* | 94.1* |
|  |  | Slight dependency (91<=BI<=99) | 28.8** | 25** | 18.4** | 90.8** | 94.3* | 95.6* |
|  |  | Independent (BI=100) | 14.7** | 15.1** | 10.5** | 93.1** | 91.2* | 94.5* |
|  | Multimorbidity | |  |  |  |  |  |  |
|  |  | Yes | 33.5* | 33.9* | 31.6 | 90.7 | 94.1* | 93.8 |
|  |  | No | 37.3* | 36.2* | 32.6 | 88.3 | 91.7* | 93.4 |
|  | Cognitive status | |  |  |  |  |  |  |
|  |  | cognitively impaired | 46.7** | 42.4** | 37.8 | 90.8 | 92.1 | 94.1 |
|  |  | Cognitively intact | 30.8** | 29.5** | 27.8 | 92.8 | 92.4 | 93.2 |
| Predisposing and enabling factors | Age | |  |  |  |  |  |  |
|  |  | Below 40 | 11.7** | 29.8** | 23.9** | 91.7 | 88.3** | 93.4 |
|  |  | 41 to 60 | 20.2** | 22.3** | 24.8** | 91.5 | 96.4** | 94.6 |
|  |  | 61 to 80 | 34.1** | 32.4** | 32.2** | 89.3 | 93.6** | 94.3 |
|  |  | over 80 | 53.5** | 53.6** | 46.6** | 87.9 | 88.8** | 89.5 |
|  | Gender | |  |  |  |  |  |  |
|  |  | Male | 27.8** | 29.3** | 27.8** | 89.1 | 93.3 | 93.5 |
|  |  | Female | 43.3** | 41.3** | 37.1** | 89.9 | 92.9 | 93.9 |
|  | Ethnicity | |  |  |  |  |  |  |
|  |  | White | 36.5** | 35.3** | 29.9** | 88.4** | 91.8 | 93.1 |
|  |  | Black Caribbean | 40.5** | 41.7** | 40.2** | 93.5** | 94.1 | 95.3 |
|  |  | Black African | 32.2** | 33.2** | 36.7** | 88.1** | 95.5 | 92.5 |
|  |  | Black other | 32.2** | 22.2** | 30** | 77.4** | 96.3 | 95 |
|  |  | Other | 26.6** | 24.8** | 20.1** | 93.1** | 93.5 | 98.3 |
|  | Relative deprivation | |  |  |  |  |  |  |
|  |  | 1st quintile | 36.8 | 34.1 | 33.6 | 91.2** | 93.4 | 92.3 |
|  |  | 2nd quintile | 34.2 | 35.1 | 31.3 | 88.1** | 93.2 | 95.5 |
|  |  | 3rd quintile | 36.2 | 38.5 | 24.1 | 85.7** | 93.3 | 97.7 |
|  |  | 4th and 5th quintiles | 32.8 | 42.1 | 28.1 | 87.1** | 92.1 | 93.7 |
|  | Education | |  |  |  |  |  |  |
|  |  | No formal education | 24.2 | 28 | 38.1 | 93.9 | 96 | 100 |
|  |  | Primary | 33.5 | 36.2 | 39.2 | 93.8 | 98.4 | 94.6 |
|  |  | Secondary | 32.2 | 33.4 | 31.1 | 92.1 | 93.2 | 93.6 |
|  |  | Tertiary | 30.3 | 28.3 | 29.8 | 90.9 | 94.3 | 91.2 |
|  | Living arrangement | |  |  |  |  |  |  |
|  |  | Private house (alone) | 54.9** | 53.3** | 46.9** | 80.5** | 84.2** | 86.5** |
|  |  | Private house (with others) | 23.9** | 23.2** | 21.6** | 94.7** | 98.2** | 97.7** |
|  |  | Sheltered home | 73.6** | 73.6** | 63.9** | 72.8** | 79.1** | 87.2** |
|  | Have someone for care for | |  |  |  |  |  |  |
|  |  | Yes | 31.9** | 33.5** | 30.6** | 91.4** | 94.4** | 94.8** |
|  |  | No | 62.5** | 66.0** | 60.5** | 62.5** | 62.2** | 57.8** |

The table shows the proportion of stroke survivors using social care and informal care under each category, if they receive any home care, as opposed to those who do not receive it. Differences between the two groups were tested using the chi-squared and the Wilcoxon Rank Sum tests. Differences are significant at: ** 1% significance level, * 5% significance level

Appendix 12: Home care use with proximity to death

|  | | ALL living in private/sheltered home | 5 year follow-up status | | | | Significance |
| --- | --- | --- | --- | --- | --- | --- | --- |
|  |  |  |  |  |  |  |  |
|  |  |  | Completed | **Died** | Missing | Not reached the follow-up |  |
| 3-month follow-up | | 3344 | 1,315  (39%) | **862**  **(26%)** | 497  (14%) | 670  (21%) |  |
|  | % received any home care at 3 months | 74.77 | 68.65 | **85.2** | 66.04 | 79.67 | P<0.03 |
|  | % received any level of social care at 3 months | 35.55 | 28.94 | **55.68** | 25.4 | 25.14 | P<0.025 |
|  | % received any level of informal care at 3 months | 89.44 | 92.68 | **88.24** | 91.43 | 84.43 | P<0.3 |
| 1-year follow-up | | 3310 | 1,476  (45%) | **673**  **(20%)** | 519  (15%) | 642  (20%) |  |
|  | % received any home care at 1 year | 73.24 | 68.72 | **85.99** | 70.5 | 72.43 | P< 0.04 |
|  | % received any level of social care at 1 year | 35.01 | 29.71 | **51.13** | 29.49 | 30.75 | P<0.06 |
|  | % received any level of informal care at 1 year | 92.99 | 92.6 | **92.2** | 94.94 | 93.33 | P<0.01 |

This table highlights the proportion of stroke survivors who have completed 3 months and 1 year follow-up and died by the 5-year follow-up and the pattern of their home care use. 20% and 26% of those who completed the 3-month follow-up died by the 1-year and 5-year follow-up respectively.

Among those who were alive at 3 months but died by the 5-year post-stroke, 85% had received some form of home care at 3 months. 56% received social care, and 88% received informal care at 3 months. These percentages are statistically significantly higher than those who completed or missed the 5-year follow-up.

Among those who were alive at 1 year but died by the 5-year post stroke, 86% had received some form of home care, 51% had received social care, and 92% had received informal care at 1 year. These percentages are statistically significantly higher than those who completed or missed the 5-year follow-up.

Appendix 13: Factors affecting home care use up to 5 years post-stroke: Complete case analysis

|  |  |  | **Receiving any home care** | **Receiving any social care if use home care** | **Receiving any informal care if use home care** |
| --- | --- | --- | --- | --- | --- |
|  | **Outcome regression: Dependent variable: Receiving home care** | |  |  |  |
|  | Follow up (ref: 3 months) | |  |  |  |
|  |  | 1 -year post-stroke | -0.039** | 0.006 | 0.019* |
|  |  | 5-year post-stroke | -0.045** | -0.023 | 0.019 |
| Need factors | Stroke severity (ref: mild stroke) | |  |  |  |
|  |  | Severe stroke | 0.075* | 0.089* | -0.011 |
|  |  | Moderate stroke | 0.020 | 0.084** | -0.009 |
|  | Level of disability (ref: Independent) | |  |  |  |
|  |  | Total dependency | 0.296** | 0.510** | -0.147** |
|  |  | Severe dependency | 0.323** | 0.397** | -0.056** |
|  |  | Moderate dependency | 0.216** | 0.164** | 0.022* |
|  |  | Slight dependency | 0.263** | 0.049* | 0.024 |
|  |  |  |  |  |  |
|  | Multimorbidity | | 0.033* | 0.012 | 0.002 |
|  | Proximity to death | | 0.038* | 0.009 | 0.006 |
| Pre-disposing and enabling factors | Age at stroke | | 0.006** | 0.004** | 0.000 |
|  | Ethnicity (ref: White) | |  |  |  |
|  |  | Black Caribbean | 0.061** | 0.071** | 0.023* |
|  |  | Black African | 0.076** | 0.061** | 0.002 |
|  |  | Black other | 0.063 | -0.051 | 0.005 |
|  |  | Other | 0.050* | -0.041 | 0.028 |
|  |  |  |  |  |  |
|  | Gender (female=1) | | 0.130** | -0.023 | 0.050** |
|  | Living arrangements (ref: living in private house alone) | |  |  |  |
|  |  | Private house (with others) | 0.236** | -0.252** | 0.124** |
|  |  | Sheltered home | 0.116** | 0.068 | 0.019 |
|  | Gender* Living arrangements | |  |  |  |
|  |  | Female* Private house (with others) | -0.139** | 0.091** | -0.040* |
|  |  | Female*Sheltered home | -0.074 | 0.077 | -0.018 |
|  |  |  |  |  |  |
|  | Have someone to turn to | | 0.130** | -0.140** | 0.290** |
|  | Relative deprivation (ref: Most deprived -1st quintile) | |  |  |  |
|  |  | 2nd quintile | -0.020 | -0.018 | -0.005 |
|  |  | 3rd quintile | -0.027 | 0.003 | -0.014 |
|  |  | 4th and 5th quintiles | -0.089* | 0.064 | -0.014 |
|  | cons |  | -0.090* | 0.334* | 0.526** |
|  |  |  |  |  |  |
| **Selection model : dependent variable : Receiving any home care use** | | |  |  |  |
|  |  | Stroke severity |  | -0.139** | |
|  |  | Barthel index |  | -0.253** | |
|  |  | multimorbidity |  | -0.540** | |
|  |  | Age at stroke |  | 0.187** | |
|  |  | Ethnicity |  | 0.031** | |
|  |  | Female |  | 0.117** | |
|  |  | Private household with others |  | 1.021** | |
|  |  | haveSomeoneToTurnORcares |  | 0.551** | |
|  |  | IMD quint |  | -0.115** | |
|  |  | diedBYnextperiod |  | 0.275** | |
|  |  | Stroke year |  | 0.007 | |
|  |  | _cons |  | -12.417 | |
| Regression diagnostics |  | var(e.IC_any) |  | 0.054 | |
|  |  | corr(e.homecareANY,e.IC_any) |  | 0.002 | |
|  |  | var(IC_any[id]) |  | 0.004 | |
|  |  | var(homecareANY[id]) |  | 0.800 | |
|  |  | corr(homecareANY[id],IC_any[id]) |  | 0.478** | |
|  | R2 Within  Between  Overall |  | 0.039  0.299  0.231 |  | |
|  | Wald chi2(23) | | 1497.8 | 459.16 | |
|  | Number of obs | | 5854 | 5854 | |
|  | Selected | |  | 4360 | |
|  | Number of groups | | 3324 |  | |

**Significant at 1% significance level *Significant at 5% significance level

The table displays the estimated coefficients obtained from complete case analysis. The outcome regressions use linear probability models with the dependent variable (1) receiving any home care, (2) receiving social care, and (3) receiving informal care. Estimating the dependent variables 2 and 3 followed the selection process, where individuals who used any home care were selected before estimating the types of care. The selection regression is a probit model with a dummy dependent variable indicating whether the SS received any home care or not. The data are cohort data. Of 8493 observations (Table 5b), only 5854 had complete data for all regression variables.

The results of the complete case analysis are similar to the results of the main analysis (Appendix 12) except for a few differences. For example, the probability of receiving any home care by severe stroke patients is higher than mild stroke patients and significant at 5%, whereas; however, in the main analysis this coefficient is insignificant., whereas the coefficient of the complete case analysis is significant at 5%. Compared to most deprived stroke survivors, those in the 2^nd^ quintile were using less home care; however, the coefficient was statistically significant only in the main analysis and not in the complete case analysis.

Appendix 14: Factors related to home care use up to 5 years post-stroke: Results from estimation using multiple imputation

|  | |  |  | **Receiving any home care** |  |
| --- | --- | --- | --- | --- | --- |
|  | |  |  |  |  |
|  | | Follow up (ref: 3 months) | |  |  |
|  | |  | 1 -year post-stroke | -0.01 |  |
|  | |  | 5-year post-stroke | -0.02 |  |
| Need factors | | Stroke severity (ref: mild stroke) | |  |  |
|  |  |  | Severe stroke | 0.06** |  |
|  |  |  | Moderate stroke | 0.02 |  |
|  |  | Level of disability (ref: Independent) | |  |  |
|  |  |  | Total dependency | 0.36** |  |
|  |  |  | Severe dependency | 0.37** |  |
|  |  |  | Moderate dependency | 0.27** |  |
|  |  |  | Slight dependency | 0.23** |  |
|  |  | Multimorbidity | | 0.04** |  |
|  |  | Proximity to death | | 0.04** |  |
| Pre-disposing and enabling factors | | Age at stroke | | 0.01** |  |
|  |  | Ethnicity (ref: White) | |  |  |
|  |  |  | Black Caribbean | 0.05** |  |
|  |  |  | Black African | 0.07** |  |
|  |  |  | Black other | 0.01 |  |
|  |  |  | Other | 0.05** |  |
|  |  | Gender (female=1) | | 0.10** |  |
|  |  | Living arrangements (ref: living in private house alone) | |  |  |
|  |  |  | Private house (with others) | 0.19** |  |
|  |  |  | Sheltered home | 0.10** |  |
|  |  | Gender * living arrangements (ref: Male* Living in private hose alone) | |  |  |
|  |  |  | Female* Private house (with others) | -0.09** |  |
|  |  |  | Female*Sheltered home | -0.04 |  |
|  |  | Have someone to turn to | | 0.10** |  |
|  |  | Relative deprivation (ref: Most deprived -1st quintile) | |  |  |
|  |  |  | 2nd quintile | -0.02 |  |
|  |  |  | 3rd quintile | -0.03 |  |
|  |  |  | 4th and 5th quintiles | -0.06** |  |
|  | | cons |  | -0.05 |  |
| Regression diagnostics | | | |  |  |
| F(29,15274.4) = 102.84  Prob > F = 0.00 | | | | |  |
| Imputations=50  Number of obs = 13345 | | | | |  |
|  |  |  |  |  |  |

**Significant at 1% significance level *Significant at 5% significance level

The table displays the estimated coefficients obtained through handling missing data using the MICE method of data imputation. Estimated outcome regression is a linear probability model with the dependent variable indicating whether the individual receives any home care.

The results with multiple imputations are similar to the results of the main analysis (Appendix 12) except for a few differences. For example, the probability of receiving any home care by severe stroke patients is higher than mild stroke patients and significant at 5%, whereas this coefficient in the main analysis coefficient is insignificant.

Appendix 15: Sensitivity analysis: Factors related to home care use (if required) and the main type of home care used (if required).

|  |  | | Home care Use (If required) | Main type of care Social care=1 |
| --- | --- | --- | --- | --- |
|  | **Follow up (ref: 3 months)** | |  |  |
|  | 1 -year post-stroke | | -0.058*** | 0.062*** |
|  | 5-year post-stroke | | -0.107*** | 0.074*** |
| Need factors | **Stroke severity (ref: mild stroke)** | |  |  |
|  | Severe stroke | | 0.076* | 0.108 |
|  | Moderate stroke | | 0.059*** | 0.071* |
|  | **Level of disability (ref: Independent)** | |  |  |
|  | Total dependency | | 0.718*** | 0.094* |
|  | Severe dependency | | 0.722*** | 0.035 |
|  | Moderate dependency | | 0.390*** | -0.063** |
|  | Slight dependency | | 0.178*** | -0.044 |
|  | **Multimorbidity** | | 0.033** | 0.005 |
|  | **Proximity to death** | | 0.100*** | -0.037 |
| Pre-disposing and enabling factors | **Age at stroke** | | 0.005*** | -0.005*** |
|  | **Ethnicity (ref: White)** | |  |  |
|  | Black Caribbean | | 0.026* | 0.057** |
|  | Black African | | 0.066*** | 0.070** |
|  | Black other | | 0.011 | 0.013 |
|  | Other | | 0.004 | -0.062 |
|  | **Gender (female=1)** | | 0.057*** | -0.027 |
|  | **Living arrangements (ref: living in private house alone)** | |  |  |
|  | Private house (with others) | | -0.004 | -0.372*** |
|  | Sheltered home | | 0.048 | 0.047 |
|  | **Gender * living arrangements (ref: Male* Living in private hose alone)** | |  |  |
|  | Female* Private house (with others) | | -0.024 | 0.044 |
|  | Female*Sheltered home | | -0.03 | 0.038 |
|  | **Have someone to turn to** | | 0.018 | -0.193*** |
|  | **Relative deprivation (ref: Most deprived -1st quintile)** | |  |  |
|  | 2nd quintile | | -0.023* | 0.014 |
|  | 3rd quintile | | -0.044* | 0.050 |
|  | 4th and 5th quintiles | | -0.021 | 0.190*** |
|  | cons |  |  |  |
| Regression diagnostics | lambda_infollowup |  | 0.230*** | -0.878*** |
|  | lambda_homecareuse |  | _ | -0.235*** |
|  | Observations | | 5163 | 2001 |
|  | R square | | 0.3675 | 0.2686 |
|  | Wald chi2 | | 2690.04 | 591.96 |

**Significant at 1% significance level *Significant at 5% significance level

This additional sensitivity analyses for objectives 2 and 3 were conducted since the SLSR questionnaire had limitations in adequately identifying home care needs and actual receipt of home care. Stroke survivors who received home care for individual IADLs did not state that they required home care in general. Therefore, we selected the stroke survivors who stated that they ‘required’ help and estimated the factors associated with it. Similarly, some stroke survivors reported their main type of home care in addition to any other home care type. Therefore, we estimated two new outcome regressions for (1) home care use (if required) and (2) the main type of home care used (if required). Both regressions used a two-step Heckman selection model. The selection regression is a probit model with a dummy dependent variable indicating (1) whether the respondent is included in the follow-up and (2) whether the respondent is included in the follow-up and used home care.

Statistically significant coefficients indicate the probability of using home care (if required) and becoming social care as the main type of home care. Appendix 16: Sensitivity analysis: Alternative approach using 10-item Barthel to identify unmet needs

|  | 3 months | 1 year | 5 year | 15 year |
| --- | --- | --- | --- | --- |
| Number of stroke survivors | 3325 | 3296 | 1829 | 490 |
| Stroke survivors who need care | 1370 (41.2%) | 1321  (40%) | 815  (44.5%) | 216  (44.08%) |
| Stroke survivors who need care and receive home care | 1242  (91%) | 1155  (87.6%) | 662  (81.3%) | 168  (77.8%) |
| Stroke survivors who need care but do not receive home care **(Unmet needs)** | 124  (9%) | 164  (12.6%) | 152  (18.7%) | 48  (22%) |
| Stroke survivors who need care and receive social care | 662  (48.5%) | 630  (47.8%) | 350  (43%) | 74  (34.3%) |
| Stroke survivors who do not need care but receive home care | 532  (27.2%) | 491  (24.9%) | 194  (19.3%) | 51  (18.5%) |
| Stroke survivors who do not need care but receive social care | 220  (11.3%) | 214  (10.8%) | 71  (7%) | 17  (6.2) |
|  | **Unmet needs by Socio-demographic and health-related differences**  **(% do not receive home care, but need care)** | | | |
| **Stroke severity** |  |  |  |  |
| Severe stroke | 5 | 0** | 3.8 |  |
| Moderate stroke | 6.1 | 5.3** | 18.9 |  |
| Mild stroke | 9.9 | 14** | 19.6 |  |
| **Functional dependency** |  |  |  |  |
| Total dependency | 3.1** | 0** | 0** |  |
| Severe dependency | 2.4** | 23** | 3.2** |  |
| Moderate dependency | 12.4** | 17.6** | 25.8** |  |
| **Multimorbidity** |  |  |  |  |
| No | 9.5 | 12.8 | 20.1 |  |
| Yes | 8.5 | 12.1 | 16.5 |  |
| **Age at stroke** |  |  |  |  |
| less than 40 | 8.7** | 11.4** | 16.7** |  |
| 40-60 | 13.9** | 19.5** | 23.3** |  |
| 61-80 | 10.1** | 13.7** | 19.4** |  |
| above 81 | 4.9** | 4.8** | 9.1** |  |
| **Gender** |  |  |  |  |
| Male | 10.9* | 14.6* | 22.2** |  |
| Female | 7.8* | 10.5* | 14.9** |  |
| **Ethnicity** |  |  |  |  |
| White | 10.4 | 13.4 | 20.6 |  |
| Black Caribbean | 8.2 | 10.3 | 14.7 |  |
| Black African | 6.2 | 14.1 | 16.5 |  |
| Black other | 5 | 16.7 | 33.3 |  |
| Other | 6.2 | 7.5 | 16.9 |  |
| **Relative deprivation (IMD)** |  |  |  |  |
| 1st quintile | 10.6 | 11.1 | 17.6 |  |
| 2nd quintile | 6.8 | 13.3 | 18.8 |  |
| 3rd quintile | 8.2 | 15.2 | 25 |  |
| 4th & 5th quintiles | 11.1 | 9.1 | 27.8 |  |
| **Living condition** |  |  |  |  |
| Private household alone | 11.4 | 14.7 | 22.1 |  |
| Private household with others | 8.2 | 11.2 | 16.3 |  |
| Sheltered home | 6.5 | 12.9 | 22.2 |  |

The table uses 10 items of the Barthel Index as sensitivity analysis, and defines ‘need’ if a stroke survivor needs help for two or more ADLs and does not receive home care(see Appendix 2). The proportion of stroke survivors with unmet needs is lower under these calculations since we considered two or more ADLs, compared to the previous approach, which counted one or more ADLs. For example, 9% of stroke survivors had unmet needs at 3 months post stroke compared to 12% in the calculations in the main analysis.

References

1. Lloyd, J., & Ross, A. (2014). The Bigger Picture: Understanding disability and care in England’s older population. *The Strategic Society Centre and Independent Age*.

2. Vlachantoni, A., Shaw, R., Willis, R., Evandrou, M., Falkingham, J., & Luff, R. (2011). Measuring unmet need for social care amongst older people. *Population Trends*, (145), 56–72. https://doi.org/10.1057/pt.2011.17

3. Dunatchik, A., Icardi, R., & Blake, M. (2019). Predicting unmet need for social care. *Journal of Long-Term Care*.

4. Andersen, R. M. (1995). Revisiting the behavioral model and access to medical care: Does it matter? *Journal of Health and Social Behavior*, 1–10.

5. Mah, J. C., Stevens, S. J., Keefe, J. M., Rockwood, K., & Andrew, M. K. (2021). Social factors influencing utilization of home care in community-dwelling older adults: A scoping review. *BMC Geriatrics*, *21*(1), 145. https://doi.org/10.1186/s12877-021-02069-1

6. Kim, B.-R., Lee, J., Sohn, M. K., Kim, D. Y., Lee, S.-G., Shin, Y.-I., Oh, G.-J., Lee, Y.-S., Joo, M. C., & Han, E. Y. (2017). Risk factors and functional impact of medical complications in stroke. *Annals of Rehabilitation Medicine*, *41*(5), 753.

7. Koller, D., Schön, G., Schäfer, I., Glaeske, G., van den Bussche, H., & Hansen, H. (2014). Multimorbidity and long-term care dependency—A five-year follow-up. *BMC Geriatrics*, *14*(1), 70.

8. Chen, L., Xiao, L. D., Chamberlain, D., & Newman, P. (2021). Enablers and barriers in hospital‐to‐home transitional care for stroke survivors and caregivers: A systematic review. *Journal of Clinical Nursing*, *30*(19–20), 2786–2807.

9. Weaver, F., Stearns, S. C., Norton, E. C., & Spector, W. (2009). Proximity to death and participation in the long‐term care market. *Health Economics*, *18*(8), 867–883.

10. Miyawaki, C. E. (2015). A review of ethnicity, culture, and acculturation among Asian caregivers of older adults (2000-2012). *Sage Open*, *5*(1), 2158244014566365.

11. Carers, U. K. (2011). Half a million voices: Improving support for BAME carers. *London: Carers UK*.

12. De Koker, B. (2009). Socio-demographic determinants of informal caregiving: Co-resident versus extra-resident care. *European Journal of Ageing*, *6*(1), 3–15.

13. Swan, L., Horgan, N. F., Fan, C. W., Warters, A., & O’Sullivan, M. (2022). Residential area socioeconomic deprivation is associated with physical dependency and polypharmacy in community-dwelling older adults: An analysis of health administrative data in Ireland. *Journal of Multidisciplinary Healthcare*, 1955–1963.

14. Quinn, M., Shepperd, S., & Floud, S. (2025). Social inequalities in the use of formal and informal home care in older women: Evidence from a large UK cohort study. *Age and Ageing*, *54*(10), afaf279. https://doi.org/10.1093/ageing/afaf279

15. Ho, I. S.-S., Mcgill, K., Malden, S., Wilson, C., Pearce, C., Kaner, E., Vines, J., Aujla, N., Lewis, S., & Restocchi, V. (2023). Examining the social networks of older adults receiving informal or formal care: A systematic review. *BMC Geriatrics*, *23*(1), 531.
